# Relative Effects of Road Risk, Habitat Suitability, and Connectivity on Wildlife Roadkills: The Case of Tawny Owls (*Strix aluco*)

**DOI:** 10.1371/journal.pone.0079967

**Published:** 2013-11-21

**Authors:** Sara M. Santos, Rui Lourenço, António Mira, Pedro Beja

**Affiliations:** 1 CIBIO - Research Center in Biodiversity and Genetic Resources, University of Évora, Évora, Portugal; 2 LabOr - Laboratory of Ornithology, Instituto de Ciências Agrárias e Ambientais Mediterrânicas, University of Évora, Évora, Portugal; 3 EDP Biodiversity Chair, CIBIO - Research Center in Biodiversity and Genetic Resources, University of Porto, Vairão, Portugal; University of Calgary, Canada

## Abstract

**Background:**

Despite its importance for reducing wildlife-vehicle collisions, there is still incomplete understanding of factors responsible for high road mortality. In particular, few empirical studies examined the idea that spatial variation in roadkills is influenced by a complex interplay between road-related factors, and species-specific habitat quality and landscape connectivity.

**Methodology/Principal Findings:**

In this study we addressed this issue, using a 7-year dataset of tawny owl (*Strix aluco*) roadkills recorded along 37 km of road in southern Portugal. We used a multi-species roadkill index as a surrogate of intrinsic road risk, and we used a Maxent distribution model to estimate habitat suitability. Landscape connectivity was estimated from least-cost paths between tawny owl territories, using habitat suitability as a resistance surface. We defined 10 alternative scenarios to compute connectivity, based on variation in potential movement patterns according to territory quality and dispersal distance thresholds. Hierarchical partitioning of a regression model indicated that independent variation in tawny owl roadkills was explained primarily by the roadkill index (70.5%) and, to a much lesser extent, by landscape connectivity (26.2%), while habitat suitability had minor effects (3.3%). Analysis of connectivity scenarios suggested that owl roadkills were primarily related to short range movements (<5 km) between high quality territories. Tawny owl roadkills were spatially autocorrelated, but the introduction of spatial filters in the regression model did not change the type and relative contribution of environmental variables.

**Conclusions:**

Overall, results suggest that road-related factors may have a dominant influence on roadkill patterns, particularly in areas like ours where habitat quality and landscape connectivity are globally high for the study species. Nevertheless, the study supported the view that functional connectivity should be incorporated whenever possible in roadkill models, as it may greatly increase their power to predict the location of roadkill hotspots.

## Introduction

Roads affect wildlife by increasing habitat fragmentation, modifying animal behaviour and movements, and increasing mortality as a consequence of road-killing [1]. The collision with vehicles is the most visible impact of roads, which in at least some circumstances, may strongly influence the size and dynamics of animal populations [2], and even result in a much larger impact on population genetic diversity than road barrier effects [3]. To reduce such impacts, a number of mitigation measures have been conceived, including for instance underpasses, fences, and warning signs [4]–[6]. Although these measures are usually expensive, they may be justified when the costs are weighed against the benefits of greatly reducing roadkills [7]. However, enhancing cost-effectiveness requires that mitigation measures are spatially limited to the most critical road sections [4], [8], [9], which demands detailed quantification of the factors responsible for roadkill hotspots [4], [10]. In particular, there is a need for developing models that accurately predict hotspot locations, which might be used during road planning, construction and exploration phases to guide the design and implementation of mitigation measures [7]. Although there is extensive research on the most important factors contributing to roadkill numbers (reviewed in [11]), few studies have used an integrative approach that evaluates the relative importance of both road-specific factors and species-specific factors when explaining roadkill spatial patterns. This distinction is important because it may imply different options and strategies in mitigation of wildlife road-mortality.

Most studies have suggested that road characteristics and the quality of the surrounding habitat play a key role in shaping roadkill patterns, e.g., [4], [8], [11]–[13]. Traffic volume is often considered the most influential road characteristic [1], [11], [12], [14], [15], along with vehicle speed and road width [11]. Other factors include fencing, embankment, and driver visibility, which frequently interact and are thus difficult to assess independently [11]. Thus, roadkill risk results from the combination of many characteristics of roads themselves. Generally, casualties increase in sections with high traffic volume or low driver visibility [11], although the relationship is not always linear, and collisions may peak in roads with intermediate traffic volume [16]. Collisions often peak also where roads cross high quality habitats, though this effect is species-specific [4], [6], [11], [14]. For instance, ungulates are more frequently killed in roads near forested areas, while amphibians and some reptiles are mostly killed near wetlands [11]. Given the recognised importance of road and habitat effects, they have been the main factors used to develop roadkill models. In many cases, however, these models have insufficient predictive ability for practical application, suggesting that additional factors may need to be considered. Increasingly, there is a perception that a greater understanding of roadkill patterns might be achieved by considering landscape factors affecting animal movement rates across roads [15], [17]. Animal movement routes are expected to concentrate along paths of least resistance [e.g., 18] that are located in sections where landscape connectivity is promoted [19]. This can lead to funnelling of movement routes through spatially delimited corridors of higher connectivity, thereby increasing the risk of collision with vehicles where movement routes intersect existing roads. Therefore, it is likely that the assessment of potential movement paths of species, and their inclusion in roadkill spatial models, might increase the predictive ability to locate roadkill hotspots. Furthermore, by considering together road characteristics, habitat suitability, and movement corridors, it might be possible to quantify the relative importance of each of these factors in shaping roadkill patterns. This is relevant, because different factors may imply different mitigation strategies and techniques to reduce wildlife road-mortality. Despite its importance, this type of modelling approach has been uncommon (but see [17], [20], [21]).

In this study we examined the relative contribution of general roadkill risk, habitat suitability and landscape functional connectivity in explaining roadkill spatial patterns of tawny owl (*Strix aluco* L.) in southern Portugal. The tawny owl is a common woodland species in Europe, including Portugal [22], [23], and is frequently reported as a traffic victim [22], [24]–[26]. These characteristics make the tawny owl a particularly adequate species to test different hypotheses about factors affecting roadkill patterns.

In order to accomplish the proposed objectives, we used a simple roadkill index based on the number of other road-killed vertebrates collected in the study area. We used detailed data on tawny owl distribution in the study area to develop habitat suitability models based on Maxent approach [27]. Also, we produced alternative functional connectivity scenarios based on least-cost path predictions (i.e., potential movement paths) between territory centroids (UNICOR; [28]). We then developed roadkill models based on Gaussian regression, and we used hierarchical partitioning to quantify the relative contribution of each set of independent variables to explain variation in tawny owl roadkills. The novel approach adopted here can probably be applied to other species and regions, and adapted to different spatial scales.

## Material and Methods

### Study Area

The study was conducted in southern Portugal, within an area of ca. 400 km^2^ (38°32′24″ to 38°47′33″N; −08°13′33″ to −07°55′45″W). The climate is Mediterranean, with mild winters and hot and dry summers. Mean annual temperature ranges from 5.8°C to 12.8°C during the winter (January), and from 16.3°C to 30.2°C during the summer (July) (Évora 1971–2000; [29]). Annual rainfall averages 609.4 mm (Évora 1971–2000; [29]). The region has an undulating relief (150 m to 400 m above sea level), and land cover is dominated (>90%) by cork oak *Quercus suber* and holm oak *Quercus rotundifolia* woodlands with varying tree density (“montado”) and agricultural areas (e.g. arable land, olive groves, vineyards).

**Figure 1:**
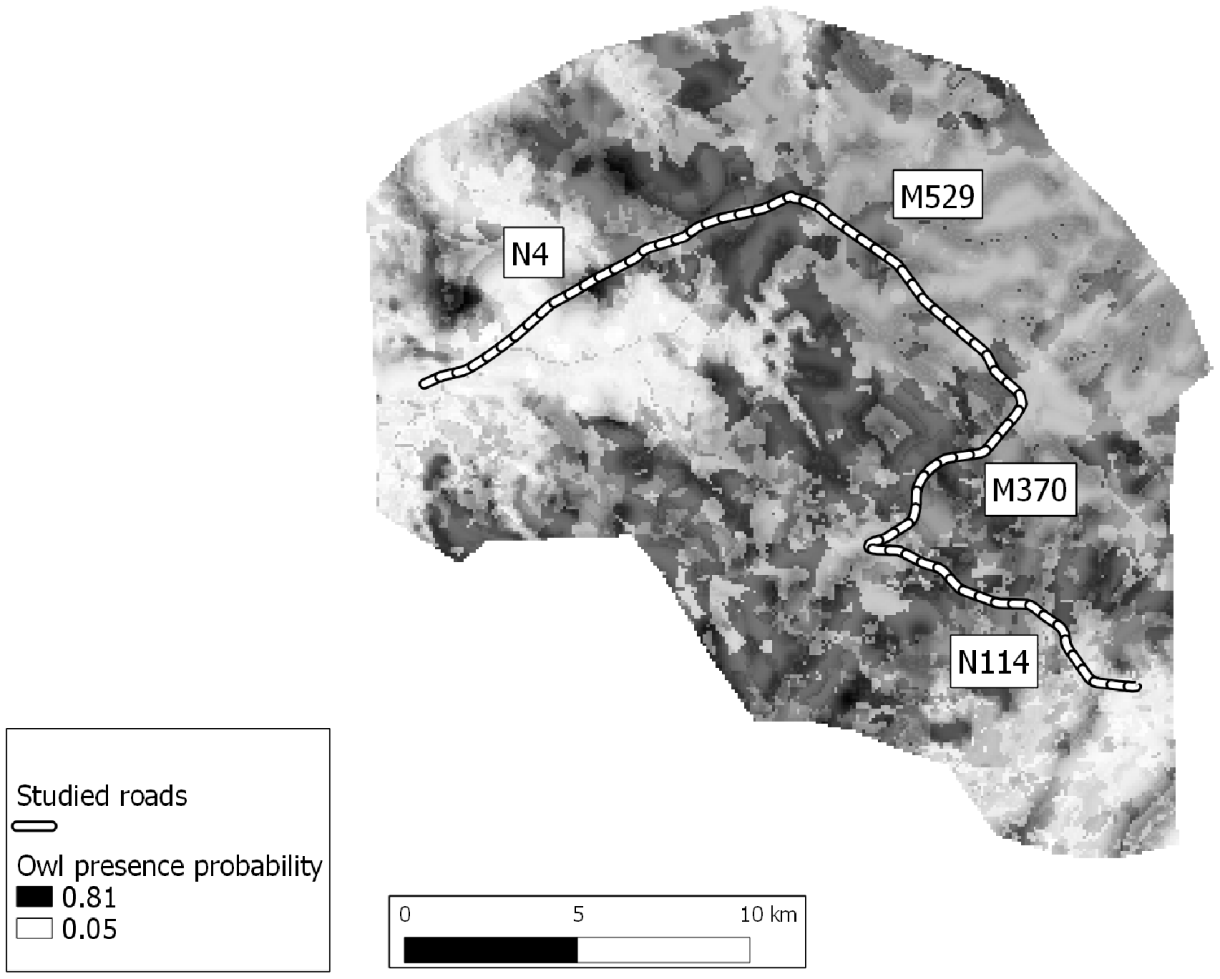
Study area and roads. Details of the study area in Portugal, with location of the four studied roads and overlay of the habitat suitability map for the tawny owl (Roads N4 and N114 are national roads, and M529 and M370 are municipal roads; darker areas in the habitat suitability map indicate higher presence probability).

Four road segments (summing to 37 km) with varying traffic volumes were selected for roadkill monitoring. Roads N4 and N114 are classified as national roads (4 000 to 10 000 vehicles/day; N114 includes road sections with more than 10 000 vehicles/day; [30]), while M529 and M370 are municipal roads (1 000 to 4 000 vehicles/day and less than 1 000 vehicles/day, respectively; [30]). All roads are two-lanes wide, without central barriers, except in two road crossings (Figure 1). The tawny owl is abundant in the oak woodlands surrounding these roads, where individuals of this species are often found dead due to collisions with vehicles [31], [32].

### Roadkill Data

We divided the studied roads in 500 m-sections, which were the units of replication for estimating factors affecting tawny owl roadkills. We collected data on all vertebrate roadkills between January 2005 and April 2012 along the four road segments on a daily (2005, 2007–2012), or weekly basis (2006). Surveys began within 2 h after sunrise and were conducted by one experienced observer driving at 20–40 km/h, and checking both sides of the road. The standard road sampling width corresponded to both lanes and shoulders (paved and unpaved). We identified every road-killed animal detected to the most precise taxonomic level, and registered its geographical coordinates with a GPS (5 m-accuracy). This procedure yielded a multi-species dataset from which we extracted the data regarding tawny owl mortality (the dependent variable) for the period between 2005 and 2012.

To estimate the intrinsic roadkill risk in each road section, we used a simple index based on the number of road-killed vertebrates collected in 2005, excluding tawny owl records. This index assumes that a section with high overall mortality also has a high intrinsic risk for tawny owls, irrespective of the number of tawny owls actually found dead in that section. This index reflects mostly the locations with higher mortality of most common species occurring in the study area. We used a single year because models in future applications should be built with easily obtainable and low-cost variables [33], and also because the sample size was adequate for the analyses. We used the multi-species roadkill index, because we wanted to reduce confounding effects of road characteristics with that of habitat suitability and movement corridors. Specifically, we have tried to control for the possibility, for instance, of road sections with characteristics potentially favouring high tawny owl mortality having in reality low mortality, just because habitat suitability in the surrounding landscape was poor and there were no movement corridors across that section. Furthermore, some important characteristics such as driver visibility, traffic volume and speed were unavailable at the scale of 500 m-road sections, thereby limiting the possibility of inferring risk from road characteristics. Therefore, we believe that this index has considerable advantages over the actual road characteristics for quantifying the intrinsic risk of each section.

### Habitat Suitability

Habitat suitability was estimated as the average probability of tawny owl occurrence within a buffer of 250 m of each 500 m-road section, which was computed using a distribution model developed for the species in the study area. The model was based on occurrence data obtained during point counts carried out in the breeding seasons (March-May) of 2005 (65 points), 2007 (68) and 2011 (75). Although we sampled most point counts in the three years, a few were sampled only once or twice due to access restrictions to private lands. Points were located at >1.2 km from each other, and they were visited after sunset, from 19∶30 to 00∶30, using playbacks of conspecific vocalizations to detect territory holders [34]. Each point was composed by 4 min of male song playback and 10 min of listening for replies (see [32]). The position of individuals responding to playbacks was registered in a 1∶25 000 topographic map and later introduced in a Geographic Information System (GIS).

We used eleven landscape variables to build the habitat model: proportional cover by nine dominant land cover types, distance to water courses and elevation. Detailed land cover maps were previously produced from GIS classification (1∶10000 scale) of digital aerial photos (2003, Associação de Municípios do Distrito de Évora), and field corrections [32]. We reclassified land cover classes into nine categories: urban, water reservoirs, riparian vegetation, open agricultural areas (dry arable lands), other agricultural areas (olive orchards, vineyards, irrigated fields), sparse (10% tree cover), medium-density (10–50% tree cover), and dense (>50% tree cover) oak woodlands, and other land cover types (pine and eucalyptus plantations, scrubland). We assessed the water courses from the land use map, and the Euclidean distance to the closest one was calculated for each pixel, creating a distance raster. Elevation was obtained from NASA (http://asterweb.jpl.nasa.gov/gdem.asp). The landscape variables were chosen due to their relevance for the species, the scale of our analysis, and their availability for the study area. Although the tawny owl is considered a woodland specialist, it also breeds in more open woodland [23], [35]. In more open areas, the riparian trees can often be occupied by owls [23], which justifies the inclusion of distance to water course and elevation variables. For habitat suitability mapping, we converted land cover variables to raster format, and all variables were limited to 90×90 m resolution.

Model development was based on presence-only approaches [e.g., 36], because it could not be assumed that owls were absent from points where they were not recorded [37], [38]. Specifically, we used the maximum entropy method (MaxEnt, [27], in which the logistic output is a continuous probability of owl occurrence ranging from 0 (unsuitable habitat) to 1 (optimal habitat), allowing its usage as a measure of habitat suitability.

We used the default values for convergence threshold (10^−4^), maximum number of interactions (500), maximum number of background points (10^4^), and regularization multiplier value (1). We considered linear, quadratic, product, threshold, and hinge transformation features [27]. We adjusted the sample radius to 3 pixels (270 m buffer). The validation of the model was performed with the bootstrap technique with 50 replicates, allowing sampling with replacement. We used the 10^th^ percentile of training presence values as logistic threshold and the area under the receiver operating curve (AUC) as threshold-dependent and -independent measures of model performance, respectively. Finally, we evaluated the importance of each environmental variable in the habitat suitability model employing the jack-knife test [27]. We used MaxEnt version 3.3.3e (http://www.cs.princeton.edu/~schapire/maxent; [27], [39].

### Connectivity Patterns

Data on actual movement patterns of tawny owl were unavailable, thus we estimated connectivity based on territory spatial distribution and habitat suitability [40]–[42]. We built a virtual map of tawny owl territories from a regular grid of 440 90-ha hexagons, corresponding to the average territory size of the species [37]. Territories with an average occupancy probability <0.40 as derived from Maxent modelling were considered vacant. We used a virtual map because the exact location of most territories was unknown, though we believe that our approach provided a reasonable approximation because field data suggested that territories were tightly packed in suitable habitat areas. We further assumed that the main movement of individuals occurred between the centroids of territories, and that movements were easier where habitat quality was higher. Because breeding tawny owls are resident year-round [24], the movements considered were judged to reflect those of dispersing juveniles and non-breeders searching for a vacant territory [43]. Estimating connectivity from habitat suitability was considered reasonable, because a telemetry study on dispersing tawny owl juveniles in Denmark showed that they had similar habitat preferences to the territorial adults [43]. The eagle owls showed the same similarities of habitat preferences [44] and it was shown that movement rates for a wide range of species tend to be greater through matrix of a more similar structure to the species’ habitat [45]. Although deviations to these patterns can introduce errors in analyses [42], we believe this is unlikely given the scarcity of tawny owls outside forested habitats in our study area.

Under the assumptions described above, we modelled the connectivity patterns using a modified Dijkstra’s algorithm, implemented in UNICOR (UNIversal CORridor and network simulation model; http://cel.dbs.umt.edu/software/UNICOR/; [28]) to find all least-cost paths (potential movement paths) between all paired combination of source and destination locations, and representing the landscape as a connectivity graph with nodes and edges [28], [46]. The surface resistance to movement was built using the probability of species presence derived from Maxent habitat modelling (movement cost = [1−presence probability]*100). We then created potential tawny owl movement routes across the study area with a Gaussian kernel density function (with linear scaling, and 5 pixels as kernel buffer window), that produces a map with the cumulative density of optimal paths buffered by a kernel density estimation. We estimated connectivity for each road section as the density of optimal paths within a 250 m-buffer.

We used 10 movement scenarios to estimate connectivity, considering two alternatives defined by territory quality, and five movement distance thresholds (1, 2, 5, and 10 km, and no distance threshold) per territory quality alternative. We estimated quality for each 90 ha-territory, from the average of predicted presence probabilities yielded by Maxent modelling. Territories with presence probabilities >0.41 were considered favourable (n = 156), whereas values >0.51 indicated high quality territories (n = 60). In one of the scenarios, movements could occur between all favourable territories, whereas movements in the other scenario were restricted to high quality territories. The later scenario was selected to account for the possibility of juvenile dispersers being produced primarily in high quality territories, which were also those most sought after by adult non-breeders. Distance thresholds were defined as Euclidean distances from territory centroids, and were used to reflect potential limits to tawny owl dispersal range.

### Data Analysis

We used Gaussian regression models to relate tawny owl roadkills per 500 m-road section to each set of explanatory variables. We specified eleven alternative models, all of which included roadkill risk (ROAD) and habitat suitability (HABITAT) variables. One of the alternative models included only these two variables, whereas the remaining 10 models included one connectivity variable at a time (estimated from each of the 10 movement scenarios; Table 1).

**Table 1:** Name, description, and summary statistics of untransformed explanatory variables (mean, standard deviation, and range values).

Variable name	Variable description	Mean ± SD	Range
ROAD^a^	Roadkill risk index (percentage of other road-killedwildlife in each 500-m road section)	1.333±0.654	0.410–4.010
HABITAT	Habitat suitability model for tawny owl(probability values)	0.377±0.146	0.09–0.600
HQ1^a^	Connectivity between high quality territories up to1 km distance (cumulative density of paths)	0.010±0.003	0–0.190
HQ2^a^	Connectivity between high quality territories up to2 km distance (cumulative density of paths)	0.063±0.170	0–0.790
HQ5^a^	Connectivity between high quality territories up to5 km distance (cumulative density of paths)	0.473±0.874	0–3.370
HQ10^a^	Connectivity between high quality territories up to10 km distance (cumulative density of paths)	2.025±3.397	0–16.530
HQ100^a^	Connectivity between high quality territories withoutdistance limit (cumulative density of paths)	3.852±7.421	0–40.240
F1^a^	Connectivity between favourable territories up to1 km distance (cumulative density of paths)	0.017±0.004	0–0.160
F2^a^	Connectivity between favourable territories up to2 km distance (cumulative density of paths)	0.206±0.304	0–1.080
F5^a^	Connectivity between favourable territories up to5 km distance (cumulative density of paths)	2.344±2.890	0–10.560
F10^a^	Connectivity between favourable territories up to10 km distance (cumulative density of paths)	12.690±0.900	0–74.050
F100^a^	Connectivity between favourable territories withoutdistance limit (cumulative density of paths)	27.800±0.377	0–216.46
SPA	Linear combination of three spatial filters obtainedfrom Spatial Eigenvector Mapping	0.000±0.647	−1.160–1.720

a transformed to power 0.3.

We powered the dependent variable to 0.5, to approach normality and remove outliers. We powered also the variable ROAD and all connectivity variables to 0.3 (Table 1). To check for collinearity between the explanatory variables, we calculated the variance inflation factors (VIF) for the 11 models. These calculations were also made to identify a possible collinearity between HABITAT and each of the connectivity variables, as resistance surfaces were obtained from habitat suitability values. As a rule of thumb, variables with VIF >10 are considered highly collinear [47].

We determined the relative support for each model using Akaike information criterion corrected for small sample sizes (AICc) and ranked all models according to three parameters: AICc differences compared with the model with lowest AICc (ΔAICc), model probabilities (*w_i_*) and evidence ratios [48]. We considered as plausible all the models with an ΔAICc <2 (when compared with the model having the lowest AICc) or with an AICc lower than the null model (no connectivity variable added). The evidence ratio was calculated relating the model probabilities of each model with the null model. This allowed us to state how better is one model relatively to another [48]. We did not perform model averaging because our aim was to evaluate the connectivity scenarios separately. Connectivity models with evidence ratio <2, compared to the null model, were not further evaluated. We designated the best model selected by AICc procedures as the ecological model.

We controlled for the effects of spatial autocorrelation by using Spatial Eigenvector Mapping (SEVM), in order to remove all significant autocorrelation from the residuals of the ecological model [49]. We incorporated a linear combination of the three most important spatial filters (those minimizing residual spatial autocorrelation) into the ecological model [50], and verified the absence of spatial autocorrelation in model residuals after these procedures. We designated this second model as the complete model. We checked the prediction power of each model by means of the Pearson correlation (*r*) between the dependent variable and fitted values. We assessed the fit of models with the residual plots and the adjusted R^2^.

We applied hierarchical variation partitioning [51], [52] to the ecological and the complete models in order to quantify the independent effects of each explanatory variable while controlling for the presence of the others, and thus identify the most likely causal factors in roadkill patterns [33], [47]. We analysed both the ecological and the complete models in order to assure that the inclusion of a spatial component did not change the relative importance of ecological variables. We used the coefficient of determination (R^2^) as a measure of variation explained by each regression model [53]. We tested the statistical significance of independent contributions of variables using a randomization procedure (Z-scores) with an upper 0.95 confidence limit [54].

We used the statistical software R version 1.14.1 [55] for building Gaussian models, and the hier.part package [56] for the partitioning analyses. We addressed the spatial autocorrelation using the routines available in the program Spatial Analysis in Macroecology (SAM, version 4.0, [57]) based on spatially explicit Gaussian regression models.

### Ethics Statements

All road-killed animals used in the present study were already found dead, and therefore an ethic or legal approval was not required. All efforts were made to minimize suffering of a few animals found still alive after being hit by a vehicle, delivering them as soon as possible to wildlife recovering centres.

About 90% of the point counts for censusing tawny owls were located in public agricultural and paved access roads and thus no permission of the owners was needed (according to the Portuguese legislation). For point counts that required entering private lands, we previously obtained oral permission from the owners to conduct the study in their properties. Whenever access was denied, we did not perform point counts inside those properties. The use of playbacks to census birds (protected species or not) in Portugal does not require any specific legal or ethics commission permission neither is referred in national legislation.

## Results

### Roadkill Patterns

During the seven-year period, we recorded 341 road-killed tawny owls (1.32 individuals/km/year; Figure S1), most of which were recorded in 2005, and 2007–2009. Numbers were lower in 2006 and 2012, when sampling was less frequent and incomplete, respectively (Figure S2). On average, we registered more tawny owls roadkills from June to August, and less from October to March. The age was determined for 39 road-killed owls, of which 56.4% were juveniles (first calendar year; [58]), and 43.6% were adults. Both age classes were found every month, except November and December. However, adults were most frequent between March and June, while most juveniles were found between April and August (Figure S3).

Specifically for the year of 2005, we recorded 4381 road-killed individuals of 107 species (birds-47.5%, amphibians-28.4%, mammals-17.7%, and reptiles-6.4%; Figure S4), representing species with different size and vagility, and including many habitat generalists (SMS, pers. observ.).

### Habitat Suitability

The tawny owl presence records (n = 339) were spatially clustered (nearest neighbour index = 0.45, Z-score = −19.55, P<0.05), probably due to the repeated records of the same individuals in the second and third visits to point counts. We tried without success several methods to reduce this pattern, and decided to use a random selection of 50% of the records (n = 169), which was still spatially clustered (nearest neighbour index = 0.48, Z-score = −13.04, P<0.05), but produced a more accurate habitat suitability map when compared to other methods.

Using the logistic threshold rule of 10^th^ percentile of training presences, the thresholds of replicated runs varied between 0.24 and 0.35, and omission rates were consistently low, with maximum value of 9%. The average training AUC for the habitat model of all replicated runs was 0.759±0.017. Both measurements indicate that the model performs better than random, and suggest that model predictions should be accurate enough to represent the realized species distribution [27]. The jack-knife analysis showed that land cover(45.3% contribution) and elevation (38.9%) were the most important variables, while distance to water had a minor contribution (15.8%). Dense and medium-density oak woodlands, and riparian vegetation were associated with tawny owl presences, while other agricultural areas were associated with absences. The species was absent from areas under 250 m elevation (figure 1).

### Connectivity Patterns

The ten connectivity scenarios covered differently the extent of the study area. The five scenarios assuming that movements occurred only between high quality territories predicted smaller extents of connected areas, when compared to the scenarios including movements between all favourable territories. As expected, allowing higher distance thresholds increased the extent of the study area predicted to be connected and reduced isolation between territories. For both scenarios of 10 and 100 km, more than half of the study area had predictions of potential movement paths (Figures S5. S6, S7, S8, S9, S10, S11, S12, S13, S14).

### Modelling Owl Roadkills

From the 11 a priori Gaussian regression models tested for tawny owl roadkill patterns, only those including the connectivity variables HQ5, HQ100, and HQ10 had higher support than the null model including only ROAD and HABITAT (Table 2). From these three models, the one including HQ5 had an empirical support 4.11 times greater than the null model, while the others were largely equivalent to the null model (evidence ratio of 1.55). Thus, the model ROAD+HABITAT+HQ5 was the best approximating ecological model, though the Akaike weight of 0.37 suggested some model selection uncertainty (Table 2). Overall, the model suggested that the number of owl roadkills was higher where the global roadkill index was also high, and where roads crossed landscapes with high connectivity. The effect of habitat suitability had an equivocal importance, as its regression coefficient was not significantly different from zero in any model (Table 3). The connectivity scenario which best fitted the roadkill data was that between high quality habitats and up to 5 km of source territories (HQ5). Moreover, connectivity between high quality territories globally received higher support than connectivity between all favourable territories (Table 2). There were no collinearity problems detected between explanatory variables: all VIF values were below 2.0 (Table 2). The spatial autocorrelation was strongly reduced in the residuals of the complete model. The residual plots revealed no other patterns, influential observations or problems with overdispersion of the data, both in the ecological and complete models.

**Table 2:** Model selection for the tawny owl roadkill data based upon Akaike information criterion (Mod #: Model number; Variables in the model: variables included in each model; df: degrees of freedom; ΔAICc: AICc differences; Model prob (*w_I_*): model probabilities; Evid ratio: evidence ratios of each model; Adj R^2^: adjusted R^2^ of each model; VIF: variance inflation factor of each model; The evidence ratio provides a measure of how better is each model relatively to the null model (model 0); see Table 1 for variable codes).

Mod #	Variables in the model	df	ΔAICc	Model probies (*w_i_*)	Evid ratio	Adj R^2^	VIF
3	ROAD+HABITAT+HQ5	5	0	0.37	4.11	0.281	1.45
5	ROAD+HABITAT+HQ100	5	1.92	0.14	1.55	0.263	1.41
4	ROAD+HABITAT+HQ10	5	1.93	0.14	1.55	0.263	1.41
0	ROAD+HABITAT	4	2.90	0.09	1.00	0.241	1.35
2	ROAD+HABITAT+HQ2	5	3.81	0.06	0.67	0.24	1.38
9	ROAD+HABITAT+F10	5	4.32	0.04	0.44	0.239	1.37
1	ROAD+HABITAT+HQ1	5	4.43	0.04	0.44	0.24	1.37
10	ROAD+HABITAT+F100	5	4.58	0.04	0.44	0.236	1.36
8	ROAD+HABITAT+F5	5	5.16	0.03	0.33	0.230	1.35
7	ROAD+HABITAT+F2	5	5.18	0.03	0.33	0.230	1.35
6	ROAD+HABITAT+F1	5	5.20	0.03	0.33	0.230	1.35

**Table 3:** Results of the ecological (EM) and the complete models (CM), and of the hierarchical partitioning applied to tawny owl roadkill data (Regression models – Coefficient: model coefficients of the explanatory variables, S.E.: standard errors, t-value: t test, p-value significance of the t test for the ecological and complete models; Hierarchical partitioning – I: independent contribution, J: joint contribution, Total: total contribution, I(%): percent independent contributions of individual variables for the explained variance of roadkill data, Z-score: statistical significance of independent contribution of variables, *p<0.05; see Table 1 for variable codes.

		Regression models				Hierarchical partitioning				
	Variables	Coefficient	S.E.	t value	p-value	I	J	Total	I (%)	Z-score
EM	Intercept	−1.457	0.838	−1.739	0.086					
	ROAD	3.129	0.727	4.301	<0.001	0.219	0.037	0.256	70.459	11.59*
	HABITAT	−0.836	0.927	−0.902	0.370	0.010	0.001	0.011	3.306	−0.180
	HQ5	0.659	0.292	2.257	0.027	0.081	0.030	0.112	26.235	3.65*
	Total					0.310				
CM	Intercept	−0.326	0.672	−0.486	0.629					
	ROAD	1.787	0.598	2.988	0.004	0.144	0.112	0.256	24.521	7.81*
	HABITAT	0.369	0.742	0.498	0.620	0.010	0.001	0.011	1.668	−0.180
	HQ5	0.310	0.233	1.332	0.187	0.057	0.055	0.112	9.614	2.38*
	SPA	0.958	0.139	6.887	<0.001	0.378	0.109	0.487	64.196	22.12*
	Total					0.589				

(ecological model: AICc = 203.3, *r = *0.557; complete model: AICc = 166.8, *r = *0.767).

### Hierarchical Partitioning

The ecological model explained 31.0% of the variance in the data set, while the complete model explained 58.9%. Most of the explained variation by each variable (in both models) was related to its independent effects (Table 3). Among the ecological variables, the roadkill risk had the highest independent contribution to explaining tawny owl mortality on roads (70.5% of the explained variance in the ecological, and 24.5% in the complete model), while connectivity accounted for 26.2% and 9.6% of the independent variation in ecological and complete models, respectively. The habitat suitability explained the least amount of data variation (3.3% and 1.7% in ecological and complete models, respectively; Table 3). Therefore, connectivity explains at least 5.7 times more independent variation than habitat suitability (complete model). The spatial variable had a substantially greater independent explanatory power over the ecological variables (64.2% in complete model), although its inclusion in the model did not substantially change the relative contribution of ecological variables. The independent contributions of variables were all statistically significant, except for habitat suitability (Table 3).

## Discussion

In this study road-specific factors appeared more important than species-specific factors in explaining roadkill patterns of tawny owl. Our findings indicate that a simple roadkill risk index for multi-species served as a valuable surrogate for predicting roadkill numbers of this species. We also found that the explanatory power of the roadkill model increased considerably when the connectivity patterns were incorporated in the predictive models of tawny owl roadkills.

### Road-related Factors

Our results confirm the importance of road-related factors in influencing the number of roadkills [e.g., 15], which can have important implications to the road monitoring programs and the design of mitigation measures. In fact, the most important ecological variable explaining the roadkill pattern of tawny owl was the roadkill risk index. Although we expected that high amounts of variance should be explained by roadkill risk, its large superiority comparatively to the other variables was unexpected. As previously explained, we used the proportion of other road-killed wildlife as a proxy for an index of roadkill danger, accounting that high numbers of other road-killed species should reflect several road characteristics that may influence owl mortality (traffic volume and speed, or road visibility, e.g. [11]). Our results showed that road sections with high numbers of tawny owl casualties are associated to sections also with high numbers of other road-killed species (mostly birds and amphibians). This can have implications to the survey of road-killed wildlife, suggesting that road sections with higher numbers of roadkills of a key-species may be a proxy for the road sections with higher numbers of fatalities of a whole local vertebrate community. This apparently points to the possibility that the mortality of a predator like the tawny owl may be used as an indicator of a wildlife roadkill hotspot. In fact, traffic volume and speed are difficult to measure accurately for large study areas, as they change continuously along road sections, days, seasons and years [59]. Our simple approach to summarize road factors may allow easy use by wildlife managers and road planners when accurate data on traffic and other road characteristics are not available.

Our results also suggest that road-specific factors could be more important than species-specific factors in explaining roadkill patterns. Although many studies have used both road- and species-specific factors (mostly habitat) when explaining patterns of roadkills, most of the approaches do not weight the importance of each factor group [4], [6], [11], [60]. In addition, some authors suggest that road-related factors are more important [15], [61], while others suggest that habitat variables are major determinants [11], [26], [62]. These contradictory results also motivated us in our present approach. Confirming the greater importance of road-specific factors relatively to species-specific also in other species would allow the application of mitigation measures to the whole community, instead of a species specific evaluation of road projects.

### Habitat Suitability

Tawny owl roadkills occurred in areas of different suitability values, and thus habitat had low power in explaining data. This lack of power may be at least partially due to the moderate precision of the habitat model in classifying owls’ presence (AUC = 0.76). Alternatively, this limited power may also indicate that some of the road-killed owls were moving in road segments of overall “low quality breeding habitats”, as evaluated through our model. Indeed, the habitat suitability values for each 500 m-road segment were derived from a 250 m-buffer area. Thus segments crossing general poor habitat areas may also include small parts of good habitat. This would also contribute to explain the apparent contradiction between the lack of influence of the habitat suitability values in roadkill patterns and the positive influence of connectivity patterns, which are modelled using the habitat map and indicate that owls move among high quality territories. One possible explanation to this difference is that, although both variables (habitat suitability and connectivity) were analysed within the 250 m-buffers, the connectivity values (density of paths) extracted for each buffer depended much on the values in the adjacent landscape, thus including “habitat” information from larger extents than the habitat suitability variable alone. Accordingly, some road sections crossing high density of predicted paths may correspond to areas of overall low quality habitat for breeding, in spite of connecting territories of high suitability. On the other hand, the differences found may also reflect different patterns of mortality for breeder adults and floaters (i.e., juveniles and non breeder adults without territories; [63]): breeder adults are killed in areas of suitable habitats (possibly within their territories), and juveniles and floaters are mostly killed on corridors of lower resistance to movement, which may include or be surrounded by less suitable habitats.

### Connectivity Patterns

Although connectivity appeared less important than road-related factors in explaining spatial variation in roadkills, it still accounted for a significant proportion of the explained variance. The connectivity pattern most supported by our data was that of displacements among high quality territories up to 5 km distance. This distance limit is in agreement with both usual movement within territories (for adults holding territories) and less frequent exploratory or dispersal movements outside territories (adults or juveniles without territories). In fact, most studies refer that movements of territorial adults outside their home range limits are infrequent, e.g., [25], [64]. On the other hand, the tawny owl is referred to display some of the shortest natal dispersal distances among the European raptors [43], and data on ringing recoveries reveals that most owls marked as juveniles (88%) settled within 5 km from the place of ringing [24]. It is possible that the casualties of territorial adults occur during small range movements (ca. 1–2 km), and mostly in individuals with territories nearby, or including the road. On the other hand, casualties of juveniles and floaters may occur during larger movements (ca. 2–5 km), corresponding mostly to individuals searching for vacant territories.

Our results also suggest that movements occur mostly between high quality territories, which can be explained by most juveniles departing from more productive territories and preferably searching for high quality vacant territories nearby (up to 5 km). In addition, there is an unknown number of floaters (adults and juveniles) living nearby territory holders (breeders) and searching for a vacant area, primarily in high quality habitats [24], [37], [38], thereby supporting our results.

The connectivity scenario best supported by the owl roadkill data should reflect the road sections with higher crossing probabilities by owls. Nevertheless, connectivity explained modest values of mortality variance. Indeed, some owls cross those road sections successfully using potential movement paths, while some are killed by vehicles. In a recent work, it was estimated that barn owls (*Tyto alba*) living next to a highway cross it 0.30 times per day, resulting in a reduced mortality risk of 0.009 [21]. For tawny owl, if most road crossings are also successful, a perfect match between potential movement paths and the roadkill pattern is not expected. In a similar approach, the spatial patterns of road crossing movements of migratory mooses (*Alces alces*) was compared with data on roadkills (for a larger study area) and the authors concluded that animal movement data alone were insufficient to predict road sections with higher mortality risk [65]. These authors suggested that road mortality increased due to road-specific characteristics (such as low light and poor road conditions) rather than to more frequent animal road crossing, which is in line with the dominant road-related factors observed in our study.

### Potential Limitations and Ways Forward

Interpretation of the results observed in our study require consideration of some potential limitations and shortcomings, though they are unlikely to affect our key conclusions. In the first place, the patterns observed may be dependent on the spatial scale and landscape context of our study, as the roadkill model was developed at a local scale and in a region where habitat fragmentation is not severe. In fact, in a study conducted at broader scale (ca. 300 km of surveyed roads) in southern Portugal, tawny owl roadkills were most related with species-specific factors, namely areas of dense oak woodland, although several road-related variables were also considered in the analyses [26]. Thus, our small-scale approach (and the use of abundance data in our models) shows the importance of considering also detailed local scale variables and population data in roadkill modelling, since associations between roadkills and environmental factors may be different from the ones gathered with larger scale studies. Similarly, Malo and colleagues [4] also found that, at a local scale, ungulate roadkills were mostly associated to road-related factors (crossroads, underpasses and guardrails) when compared to larger scales, thereby supporting our general results for small scale extents. Our results are however relevant to conservation practitioners, since mitigation measures of most road infrastructures also have a local scope.

The ecological variables used here also deserve some comments. On the one hand, the use of a roadkill risk index of multi-species as an effective proxy for tawny owl mortality should be still validated within a larger study area and greater landscape diversity. On the other hand, our roadkill risk variable may also reflect the attraction to roads by some owls to feed on carcasses of small animals, which may increase the risk of an owl being killed by a vehicle. However, this carrion is more abundant, according to our definition, in high roadkill risk sections and thus this part of owl mortality is accounted for in our models with the roadkill risk variable.

Despite a possible effect of differential road mortality of territory owners and juveniles/floaters, the habitat suitability model could be improved by adding additional explanatory variables to the maximum entropy model. Microhabitat descriptors (e.g., distance to hedgerows, vegetation structure of road verges, availability of hunting perches, etc.) could have increased the explanatory power of habitat suitability in our roadkill model. However, we are aware that microhabitat information is not available for large spatial scales. Alternatively, using owl abundance data, instead of presence, could have resulted in a model with higher precision, as the tawny owl is abundant and widespread in our study area. Some authors defend that distribution models of common/generalist species have lower power and classification accuracies when compared to more rare/specialist species [66].

Another potential problem is that the connectivity patterns were estimated assuming that movement rates of owls are greater in habitats of lower resistance and that breeding habitat suitability is an adequate surrogate of that resistance. Recently, there has been some debate on the use of resource selection functions to derive resistance surfaces, which imply that patterns of habitat use within home ranges are similar from patterns during dispersal movements [42], [67]. In our study, road-killed owls included both territory holders and floaters, and thus the movement models referred to both movement types: within home ranges and dispersal movements. The connectivity variable represents the patterns of habitat use for the whole population, including breeding and non-breeding individuals. In a strongly territorial species, such as the tawny owl, breeding individuals are much restricted to their home ranges. Also, non-breeders spend most of their time searching for mates and vacant territories with good breeding habitat where they can establish themselves [43], which suggest that they should occur primarily in habitats that are favourable for breeding. According to this, we considered that using the best available information on habitat use (although it refers only to breeding habitat) was a reasonable assumption to build a connectivity model for our study area, as done in many other studies or species with very different ecological requirements [40], [41], [68]–[70].

To the best of our knowledge, this is the first study to simultaneously evaluate the relative importance of roadkill risk, habitat suitability and connectivity patterns on wildlife road mortality data. Particularly, specific research on the inclusion of connectivity simulations as predictors of roadkill abundance is still rare. In addition, the present work also uses a simple index of multi-species mortality on the road to express the roadkill risk for a single species, which may be of high usefulness for assessing roadkill risk of rare and endangered species based on overall roadkill data.

Our results also raise new important questions to be addressed in future studies. The use of a roadkill index should be further explored and validated against other potential indicator species. For instance, it is known that small bodied size animals are frequently underestimated in road samplings [71]. Thus, can numbers and location of large and/or generalist species casualties, that are easier to sample, be a proxy for numbers and location of small/specialist species mortality on the road ? On what concerns the tawny owl, connectivity modelling and dispersal movements should be further addressed with telemetry data and assessment of individual responses to the roads. In addition, the consequences of tawny owl road mortality on population dynamics need to be evaluated. If most dispersal movements of tawny owls are within short distances, to what extent does the presence of roads influence population demographic structure? Future studies should find the answers to these questions.

## Supporting Information

Figure S1 **Spatial distribution of abundance of tawny owl roadkills in the study area, overlaid with main land uses (white: water reservoir, light grey: agricultural and open areas; dark grey: “montado” and other forests, crossed white: urban areas).**
(TIF)

Figure S2 **Total numbers of tawny owls road-killed in the study area, per month and for each year separately (2005–2012; n = 341).**
(TIFF)

Figure S3 **Numbers of adults and juveniles of tawny owl road-killed in the study area through the year (2005–2012; n = 39).**
(TIFF)

Figure S4 **Spatial distribution of the values of roadkill risk index (percentage of general fauna road-killed in each 500 m section) in the study area, overlaid with main land uses (white: water reservoir, light grey: agricultural and open areas; dark grey: “montado” and other forests, crossed white: urban areas).**
(TIF)

Figure S5 **Connectivity model for a pattern of connectivity among high quality territories up to 1 km distance (HQ1), overlaid with owl mortality (lighter areas indicate higher movement probability).**
(TIF)

Figure S6 **Connectivity model for a pattern of connectivity among high quality territories up to 2 km distance (HQ2), overlaid with owl mortality (lighter areas indicate higher movement probability).**
(TIF)

Figure S7 **Connectivity model for a pattern of connectivity among high quality territories up to 5 km distance (HQ5), overlaid with owl mortality (lighter areas indicate higher movement probability).**
(TIF)

Figure S8 **Connectivity model for a pattern of connectivity among high quality territories up to 10 km distance (HQ10), overlaid with owl mortality (lighter areas indicate higher movement probability).**
(TIF)

Figure S9 **Connectivity model for a pattern of connectivity among high quality territories up to 100 km distance (HQ100), overlaid with owl mortality (lighter areas indicate higher movement probability).**
(TIF)

Figure S10 **Connectivity model for a pattern of connectivity among favourable territories up to 1 km distance (F1), overlaid with owl mortality (lighter areas indicate higher movement probability).**
(TIF)

Figure S11 **Connectivity model for a pattern of connectivity among favourable territories up to 2 km distance (F2), overlaid with owl mortality (lighter areas indicate higher movement probability).**
(TIF)

Figure S12 **Connectivity model for a pattern of connectivity among favourable territories up to 5 km distance (F5), overlaid with owl mortality (lighter areas indicate higher movement probability).**
(TIF)

Figure S13 **Connectivity model for a pattern of connectivity among favourable territories up to 10 km distance (F10), overlaid with owl mortality (lighter areas indicate higher movement probability).**
(TIF)

Figure S14 **Connectivity model for a pattern of connectivity among favourable territories up to 100 km distance (F100), overlaid with owl mortality (lighter areas indicate higher movement probability).**
(TIF)

## References

[1] Seiler A (2001) Ecological Effects of Roads: A review. Introductory Research Essay No 9, Department of Conservation Biology: University of Agricultural Sciences, S-730 91 Riddarhyttan, Sweden.

[2] Fahrig L, Rytwinski T (2009) Effects of roads on animal abundance: an empirical review and synthesis. Ecol Soc 14: 1–20.

[3] Jackson ND, Fahrig L (2011) Relative effects of road mortality and decreased connectivity on population genetic diversity. Biol Conserv 144: 3143–3148.

[4] Malo JE, Suárez F, Díez A (2004) Can we mitigate animal-vehicle accidents using predictive models? J Appl Ecol 41: 701–710.

[5] Ramp D, Caldwell J, Edwards K, Warton D, Croft D (2005) Modelling of wildlife fatality hotspots along the snowy mountain highway in New South Wales, Australia. Biol Conserv 126: 474–490.

[6] Grilo C, Bissonette J, Santos-Reis M (2009) Spatial-temporal patterns in Mediterranean carnivore road casualties: Consequences for mitigation. Biol Conserv 142: 301–31.

[7] Huijser M, Duffield J W, Clevenger AP, Ament RJ, McGowen PT (2009) Cost–benefit analyses of mitigation measures aimed at reducing collisions with large ungulates in the United States and Canada; a decision support tool. Ecol Soc 14: 15.

[8] Clevenger AP, Chruszcz B, Gunson K (2003) Spatial patterns and factors influencing small vertebrate fauna road-kill aggregations. Biol Conserv 109: 15–26.

[9] Clevenger AP, Waltho N (2005) Performance indices to identify attributes of highway crossing structures facilitating movement of large mammals. Biol Conserv 121: 453–464.

[10] Jaarsma CF, Langevelde FV, Baveco JM, Eupen MV, Arisz J (2007) Model for rural transportation planning considering simulating mobility and traffic kills in the badger *Meles meles* . Ecol Inform 2: 73–82.

[11] Gunson KE, Mountrakis G, Quackenbush LJ (2011) Spatial wildlife–vehicle collision models: a review of current work and its application to transportation mitigation projects. J Environ Manage 92: 1074–1082. 21190788 10.1016/j.jenvman.2010.11.027

[12] Fahrig L, Pedlar JH, Pope SE, Taylor PD, Wegner J F (1995) Effect of road traffic on amphibian density. Biol Conserv 74: 177–182.

[13] Trombulak SC, Frissell C (2000) Review of ecological effects of roads on terrestrial and aquatic communities. Conserv Biol 14: 18–30.

[14] Erritzoe J, Mazgajski T D, Rejt L (2003) Bird casualties on European roads- a review. Acta Ornithol 38: 77–93.

[15] Litvaitis JA, Tash JP (2008) An Approach Toward Understanding Wildlife-Vehicle Collisions. Environ Manage 42: 688–697. 18427884 10.1007/s00267-008-9108-4

[16] Seiler A (2005) Predicting locations of moose–vehicle collisions in Sweden. J Appl Ecol 42: 371–382.

[17] Lesbarrères D, Fahrig L (2012) Measures to reduce population fragmentation by roads: what has worked and how do we know? Trends Ecol Evol 1514: 1–7. 10.1016/j.tree.2012.01.015 22356922

[18] Adriaensen F, Chardon JP, De Blust G, Swinnen E, Villalba S, et al (2003) The application of ‘least-cost’ modelling as a functional landscape model. Landscape Urban Plan 64: 233–247.

[19] Taylor PD, Fahrig L, Henein K, Merriam G (1993) Connectivity is a vital element of landscape structure. Oikos 68: 571–573.

[20] Grilo C, Ascensão F, Santos-Reis M, Bissonette J (2011) Do well-connected landscapes promote road-related mortality? Eur J Wildlife Res 57: 707–716.

[21] Grilo C, Sousa J, Ascensão F, Matos H, Leitão I, et al (2012) Individual Spatial Responses towards Roads: Implications for Mortality Risk. PLoS ONE 7(9): e43811 doi:10.1371/journal.pone.0043811 22970143 10.1371/journal.pone.0043811 PMC3435373

[22] Mikkola H (1983) Owls of Europe. T & A.D Poyser: Calton.

[23] Equipa Atlas (2008) Atlas das Aves Nidificantes em Portugal (1999–2005). Instituto da Conservação da Natureza e da Biodiversidade, Sociedade Portuguesa para o Estudo das Aves, Parque Natural da Madeira e Secretaria Regional do Ambiente e do Mar: Assírio & Alvim, Lisboa.

[24] Hirons GJM (1985) The effects of territorial beahaviour on the stability and dispersion of Tawny owl (*Strix aluco*) populations. J Zool, Lond (B) 1: 21–48.

[25] Baudvin H, Jouaire S (2003) Les causes de mortalité chez les chouettes hulottes adultes *Strix aluco* dans quelques forêts de Bourgogne. Alauda 71: 221–226.

[26] Gomes L, Grilo C, Silva C, Mira A (2009) Identification methods and deterministic factors of owl roadkill hotspot locations in Mediterranean landscapes. Ecol Res 24: 355–370.

[27] Phillips SJ, Anderson RP, Schapire RE (2006) Maximum entropy modeling of species geographic distributions. Ecol Model 190: 231–259.

[28] Landguth EL, Hand BK, Glassy J, Cushmann SA, Sawaya MA (2011) UNICOR: a species connectivity and corridor network simulator. Ecography 34: 1–6.

[29] IM (2010) Instituto de Metereologia, IP, Portugal (www.meteo.pt) [assessed 2^nd^ July 2012].

[30] EP (2005) Recenseamento do tráfego – Évora. Estradas de Portugal, E.P.E.

[31] Pereira M, Lourenço R, Mira A (2011) The role of habitat connectivity on road mortality of tawny owls. GeoFocus 11: 70–90.

[32] Silva CC, Lourenço R, Godinho S, Gomes E, Sabino-Marques H, et al (2012) Major roads have a negative impact on the Tawny Owl *Strix aluco* and the Little Owl *Athene noctua* populations. Acta Ornithol 47: 47–54.

[33] Mac Nally R (2000) Regression and model-building in conservation biology, biogeography and ecology: the distinction between – and reconciliation of - “predictive” and “explanatory” models. Biodivers Conserv 9: 655–671.

[34] Redpath SM (1994) Censusing tawny owls *Strix aluco* using imitation calls. Bird Study 41: 192–198.

[35] Zuberogoitia I, Martínez-Climent JA (2003) Cárabo Común, *Strix aluco.* In: Martí R, del Moral JC, editors. Atlas de las Aves Reproductoras de España, Dirección General de Conservación de la Naturaleza – Sociedad Española de Ornitologia: Madrid.pp. 320–321.

[36] Elith J, Graham CH, Anderson RP, Dudík M, Ferrier S, et al (2006) Novel methods improve prediction of species’ distributions from occurrence data. Ecography 29: 129–151.

[37] Sunde P, Bølstad M (2004) A telemetry study of the social organization of a tawny owl (*Strix aluco*) population. J Zool 263: 65–76.

[38] Campioni L, Delgado MM (2010) PenterianiV (2010) Social status influences microhabitat selection: breeder and floater Eagle Owls *Bubo bubo* use different post sites. Ibis 152: 569–579.

[39] Phillips SJ, Dudík M (2008) Modeling of species distributions with Maxent: new extensions and a comprehensive evaluation. Ecography 31: 161–175.

[40] Chetkiewicz CLB, Boyce MS (2009) Use of resource selection functions to identify conservation corridors. J Applied Ecol 46, 1036–1047.

[41] Walpole AA, Bowman J, Murray DL, Wilson PJ (2012) Functional connectivity of lynx at their southern range periphery in Ontario, Canada. Landsc Ecol 27: 761–773.

[42] Zeller KA, McGarigal K, Whiteley AR (2012) Estimating landscape resistance to movement: a review. Landsc Ecol 27: 777–797.

[43] Sunde P (2011) What do we know about territorial behaviour and its consequences in tawny owls? In Zuberogoitia I., Martínez J.E. (ed) 2011. Ecology and Conservation of european forest-dwelling raptors. Departamento de Agricultura de la Diputación Foral de Bizkaia, Estudios Medioambientales Icarus S.L.

[44] Delgado MM, Penteriani V, Nams VO, Campioni L (2009) Changes of movement patterns from early dispersal to settlement. Behav Ecol Sociobiol 64: 35–43.

[45] Eycott AE, Stewart GB, Buyung-Ali LM, Bowler DE, Watts K, et al (2012) A meta-analysis on the impact of different matrix structures on species movement rates. Landsc Ecol 27: 1263–1278.

[46] Minor ES, Urban DL (2008) A Graph-Theory Framework for Evaluating Landscape Connectivity and Conservation Planning. Conserv Biol 22: 297–307. 18241238 10.1111/j.1523-1739.2007.00871.x

[47] Zuur AF, Ieno EN, Smith GM (2007) Analysing Ecological Data. Springer Science: New York.

[48] Burnham KP, Anderson DR (2002) Model selection and multimodel inference: a practical information-theoretic approach. Springer Verlag: New York.

[49] Griffith DA, Peres-Neto PR (2006) Spatial modeling in ecology: the flexibility of eigenfunction spatial analyses. Ecology 87: 2603–2613. 17089668 10.1890/0012-9658(2006)87[2603:smietf]2.0.co;2

[50] Diniz-Filho JAF, Bini LM (2005) Modelling geographical patterns in species richness using eigenvector-based spatial filters. Global Ecol Biogeogr 14: 177–185.

[51] Chevan A, Sutherland M (1991) Hierarchical Partitioning. Am Sta 45: 90–96.

[52] Mac Nally R (1996) Hierarchical partitioning as an interpretative tool in multivariate inference. Aust J Ecol 21: 224–228.

[53] Quinn GP, Keough MJ (2002) Experimental Design and Data Analysis for Biologists. Cambridge University Press: Cambridge.

[54] Mac Nally R (2002) Multiple regression and inference in ecology and conservation biology: further comments on identifying important predictor variables. Biodivers Conserv 11: 1397–1401.

[55] R Development Core Team (2011) R: A language and environment for statistical computing. R Foundation for Statistical Computing, Vienna, Austria. Available from http://www.R-project.org.

[56] Walsh C, Mac Nally R (2008) The hier.part package. Hierarchical Partitioning. R project for statistical computing. Available from http://cran.r-project.org/.

[57] Rangel TF, Diniz-Filho JAF, Bini LM (2010) SAM: a comprehensive application for Spatial Analysis in Macroecology. Ecography 33 46–50: 50.

[58] Martínez JA, Zuberogoitia I, Alonso R (2002) Rapaces Nocturnas, Guía para la determinación de la edad y el sexo en las estrigiformes ibéricas. Monticola Ed.: Madrid.

[59] Bissonette J, Kassar KA (2008) Locations of Deer–vehicle Collisions are Unrelated to Traffic Volume or Posted Speed Limit. Human–Wildlife Conflicts 2: 122–130.

[60] Kolowski JM, Nielsen CK (2008) Using Penrose distance to identify potential risk of wildlife–vehicle collisions. Biol Conserv 141: 1119–1128.

[61] Colino-Rabanal VJ, Lizana M, Peris SJ (2011) Factors influencing wolf *Canis lupus* roadkills in Northwest Spain. Eur J Wildlife Res 57: 399–409.

[62] Roger E, Ramp D (2009) Incorporating habitat use in models of fauna fatalities on roads. Divers Distrib 15: 222–231.

[63] Penteriani V, Ferrer M, Delgado MM (2011) Floater strategies and dynamics in birds, and their importance in conservation biology: towards an understanding of nonbreeders in avian populations. Anim Conserv 14: 233–241 doi:–10.1111/j.1469–1795.2010.00433.x

[64] Martin G (1990) Birds by night. T & AD Poyser: London.

[65] Neumann W, Ericsson G, Dettki H, Bunnefeld N, Keuler NS, cet al (2012) Difference in spatiotemporal patterns of wildlife road-crossings and wildlife-vehicle collisions. Biol Conserv 145: 70–78.

[66] Hernandez PA, Graham CH, Master L, Albert D (2006) The effect of sample size and species characteristics on performance of different species distribution modeling methods. Ecography 29: 773–785.

[67] Spear SF, Balkenhol N, Fortin M-J, McRae BH, Scribner KM (2010) Use of resistance surfaces for landscape genetic studies: considerations for parameterization and analysis. Mol Ecol 19: 3576–3591. 20723064 10.1111/j.1365-294X.2010.04657.x

[68] O’Brien D, Manseau M, Fall A Fortin M-J (2006) Testing the importance of spatial configuration of winter habitat for woodland caribou: An application of graph theory. Biol Conserv 2006: 130, 70–83.

[69] Wang Y, Yang K, Bridgman CL, Lin L (2008) Habitat suitability modeling to correlate gene flow with landscape connectivity. Landsc Ecol 23: 989–1000.

[70] Huck M, Jedrzejewski W, Borowik T, Jedrzejewska B, Nowak S, et al (2011) Analyses of least cost paths for determining effects of habitat types on landscape permeability: wolves in Poland. Acta Theriol 56: 91–101. 21350594 10.1007/s13364-010-0006-9 PMC3026926

[71] Santos SM, Carvalho F, Mira A (2011) How Long Do the Dead Survive on the Road? Carcass Persistence Probability and Implications for Road-Kill Monitoring Surveys. PLoS ONE 6: e25383. 21980437 10.1371/journal.pone.0025383 PMC3181337

