# Supplementary figures and images for: Relative Effects of Road Risk, Habitat Suitability, and Connectivity on Wildlife Roadkills: The Case of Tawny Owls (Strix aluco)
Source: PLoS One. 2013 Nov 21;8(11):e79967. doi: 10.1371/journal.pone.0079967 (PMC3836987; doi:10.1371/journal.pone.0079967)

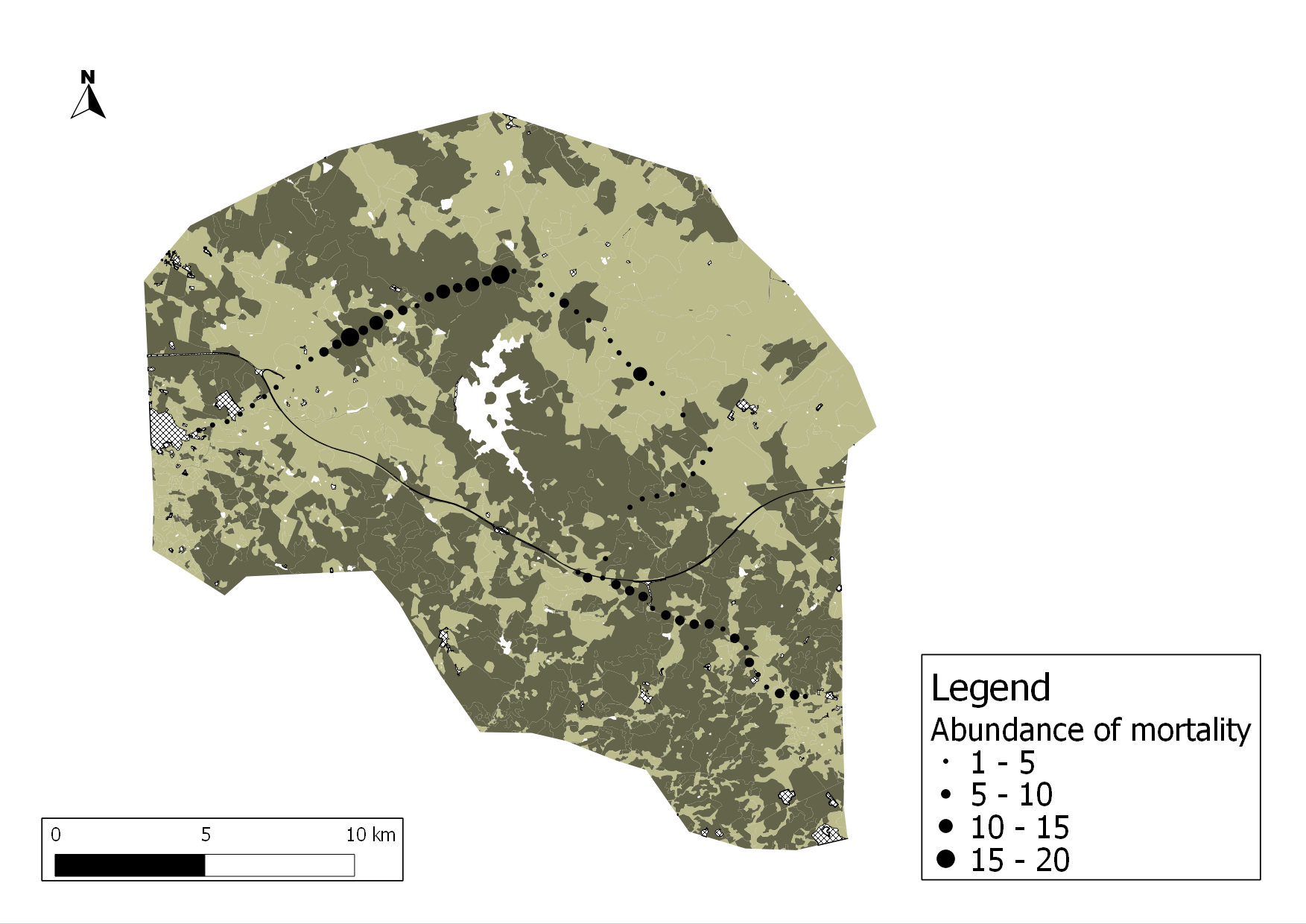

Supplement: Figure S1 — Spatial distribution of abundance of tawny owl roadkills in the study area, overlaid with main land uses (white: water reservoir, light grey: agricultural and open areas; dark grey: “montado” and other forests, crossed white: urban areas). (TIF) [file pone.0079967.s001.tif]

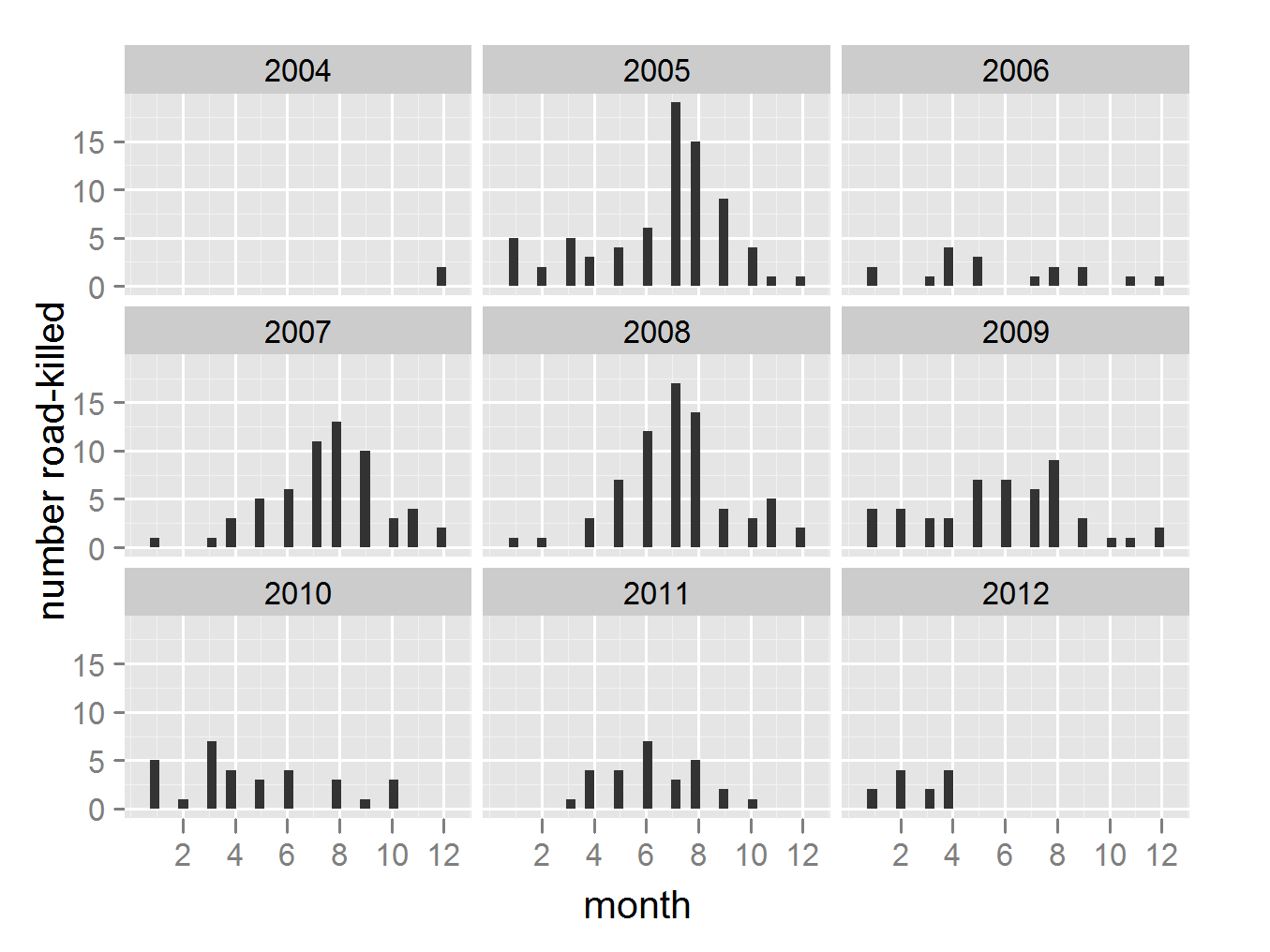

Supplement: Figure S2 — Total numbers of tawny owls road-killed in the study area, per month and for each year separately (2005–2012; n = 341). (TIFF) [file pone.0079967.s002.tiff]

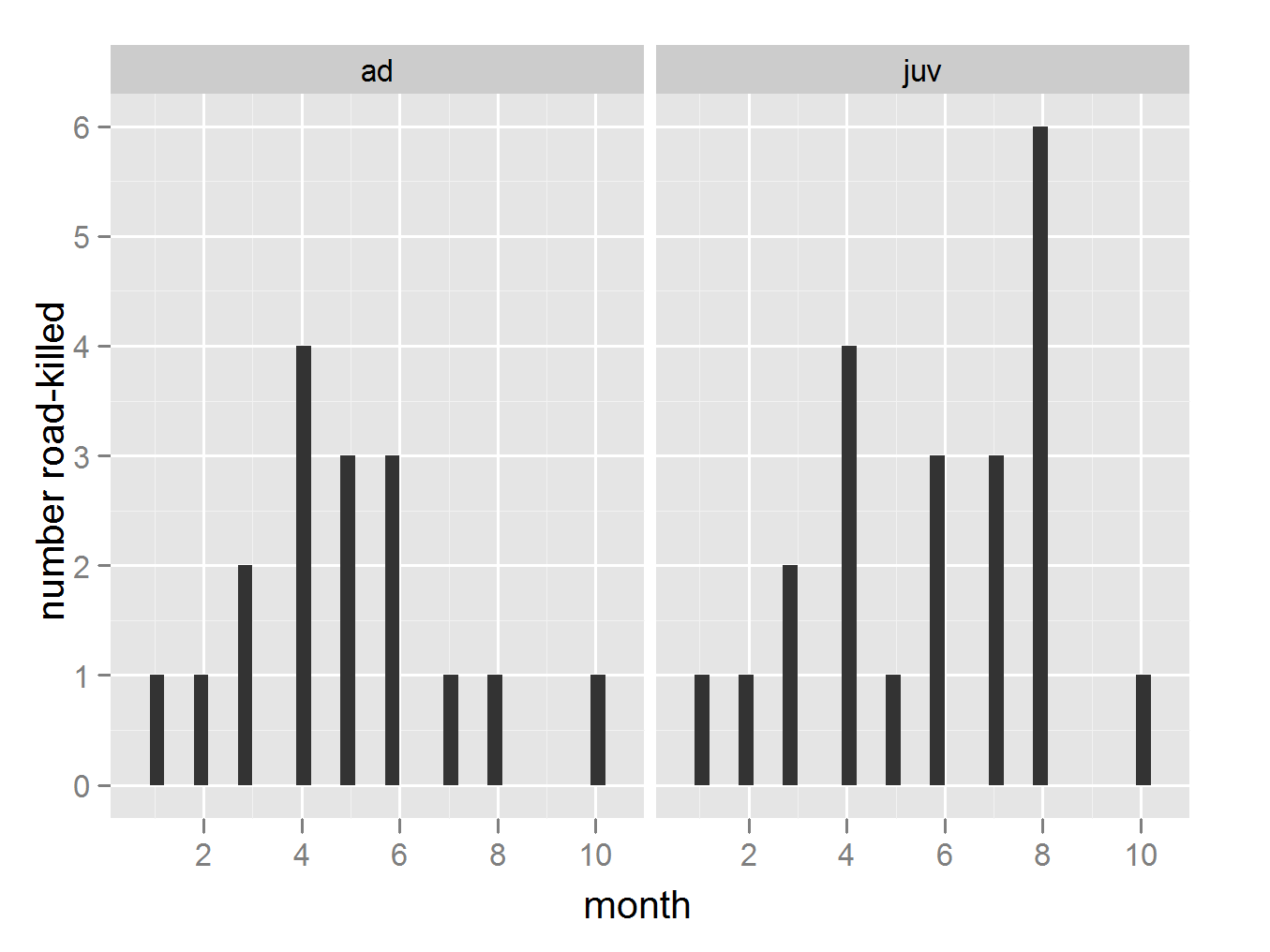

Supplement: Figure S3 — Numbers of adults and juveniles of tawny owl road-killed in the study area through the year (2005–2012; n = 39). (TIFF) [file pone.0079967.s003.tiff]

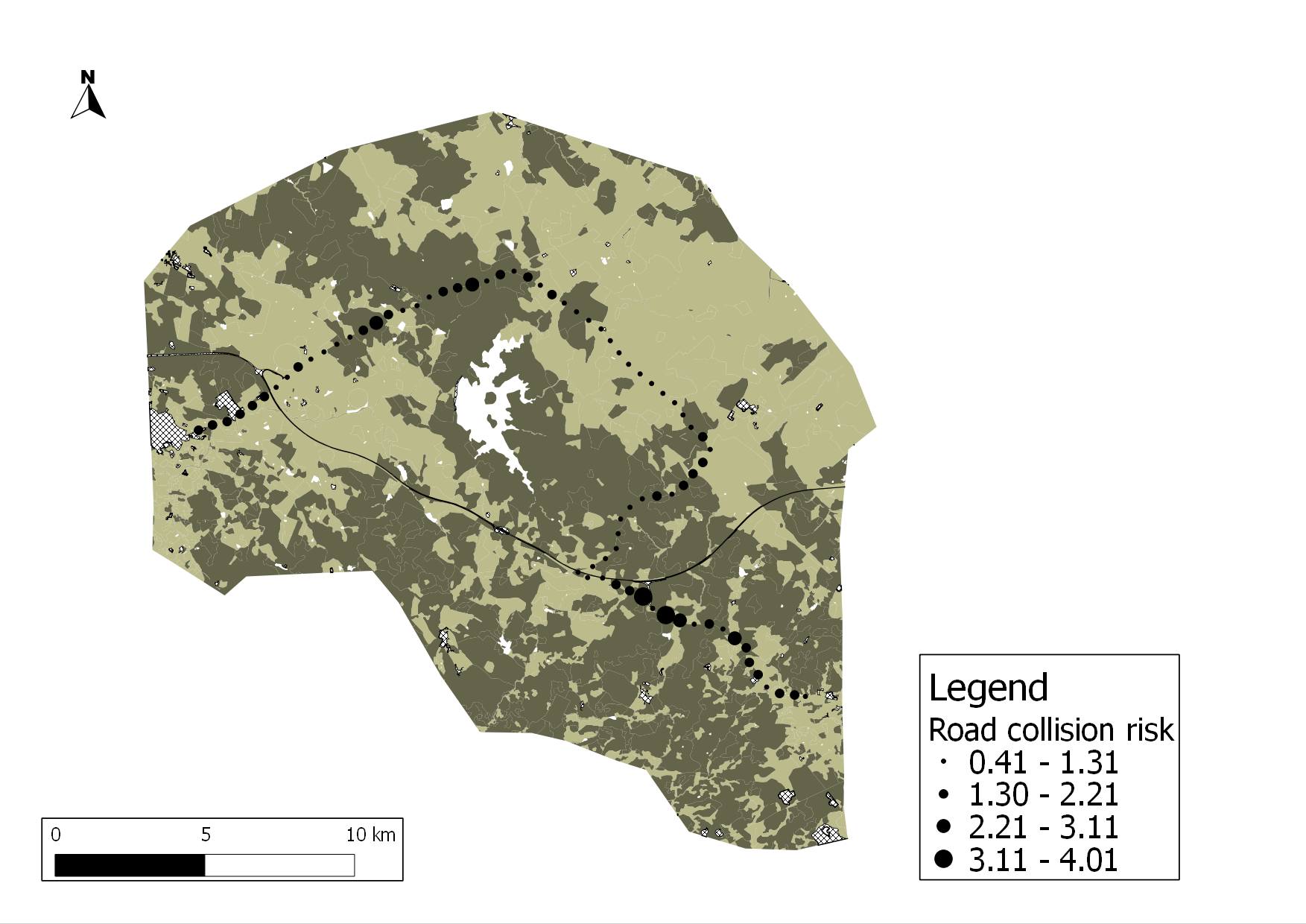

Supplement: Figure S4 — Spatial distribution of the values of roadkill risk index (percentage of general fauna road-killed in each 500 m section) in the study area, overlaid with main land uses (white: water reservoir, light grey: agricultural and open areas; dark grey: “montado” and other forests, crossed white: urban areas). (TIF) [file pone.0079967.s004.tif]

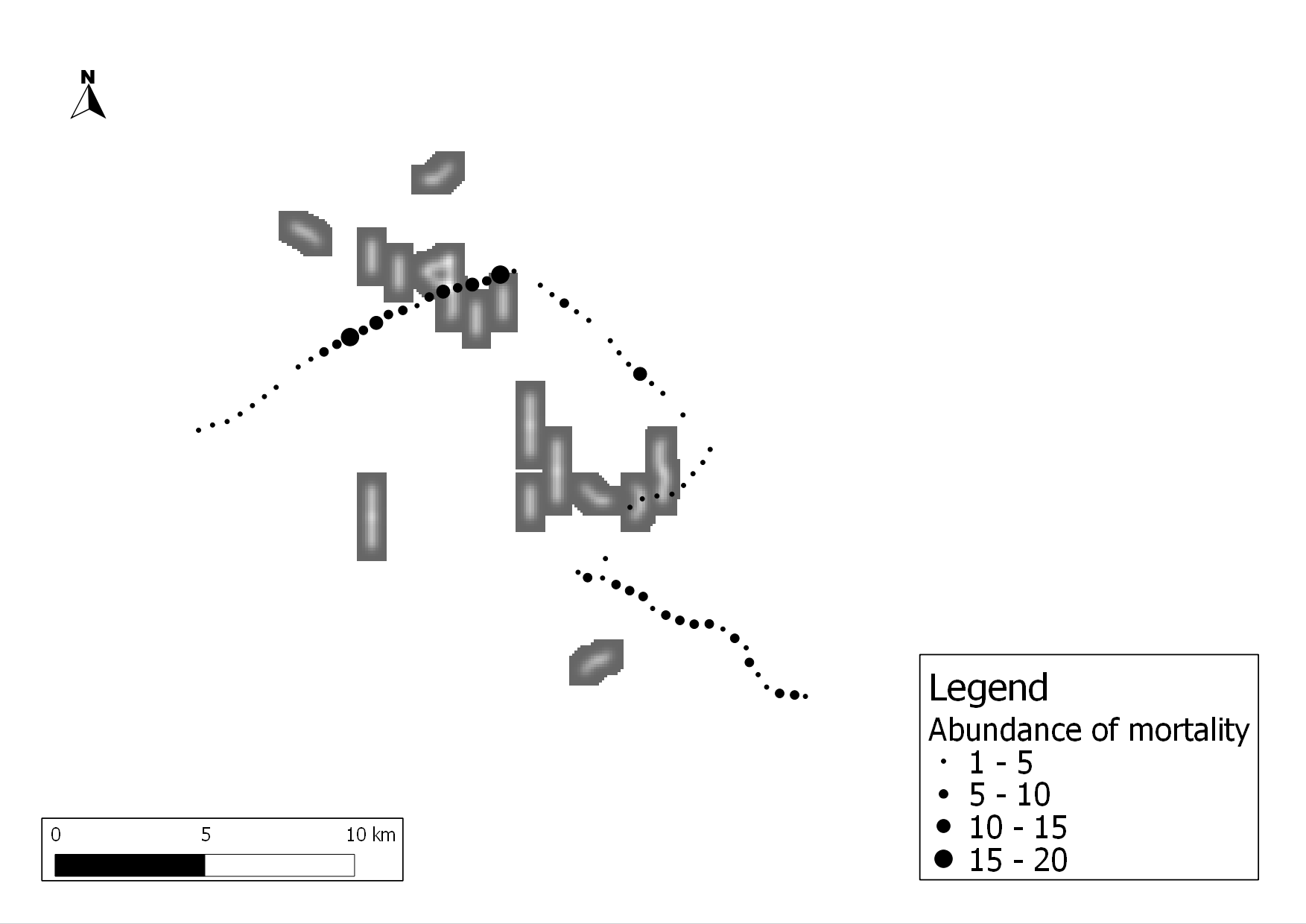

Supplement: Figure S5 — Connectivity model for a pattern of connectivity among high quality territories up to 1 km distance (HQ1), overlaid with owl mortality (lighter areas indicate higher movement probability). (TIF) [file pone.0079967.s005.tif]

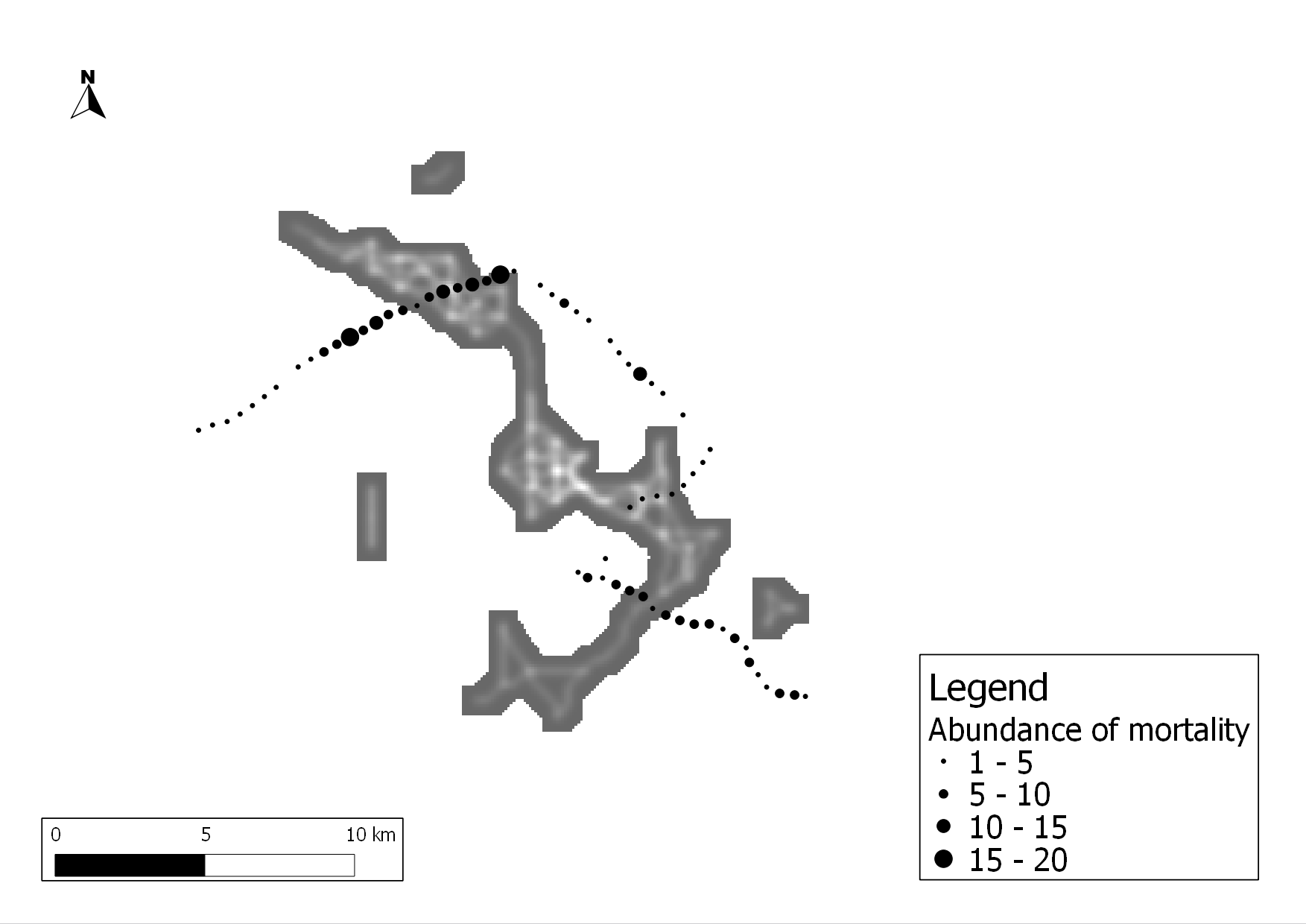

Supplement: Figure S6 — Connectivity model for a pattern of connectivity among high quality territories up to 2 km distance (HQ2), overlaid with owl mortality (lighter areas indicate higher movement probability). (TIF) [file pone.0079967.s006.tif]

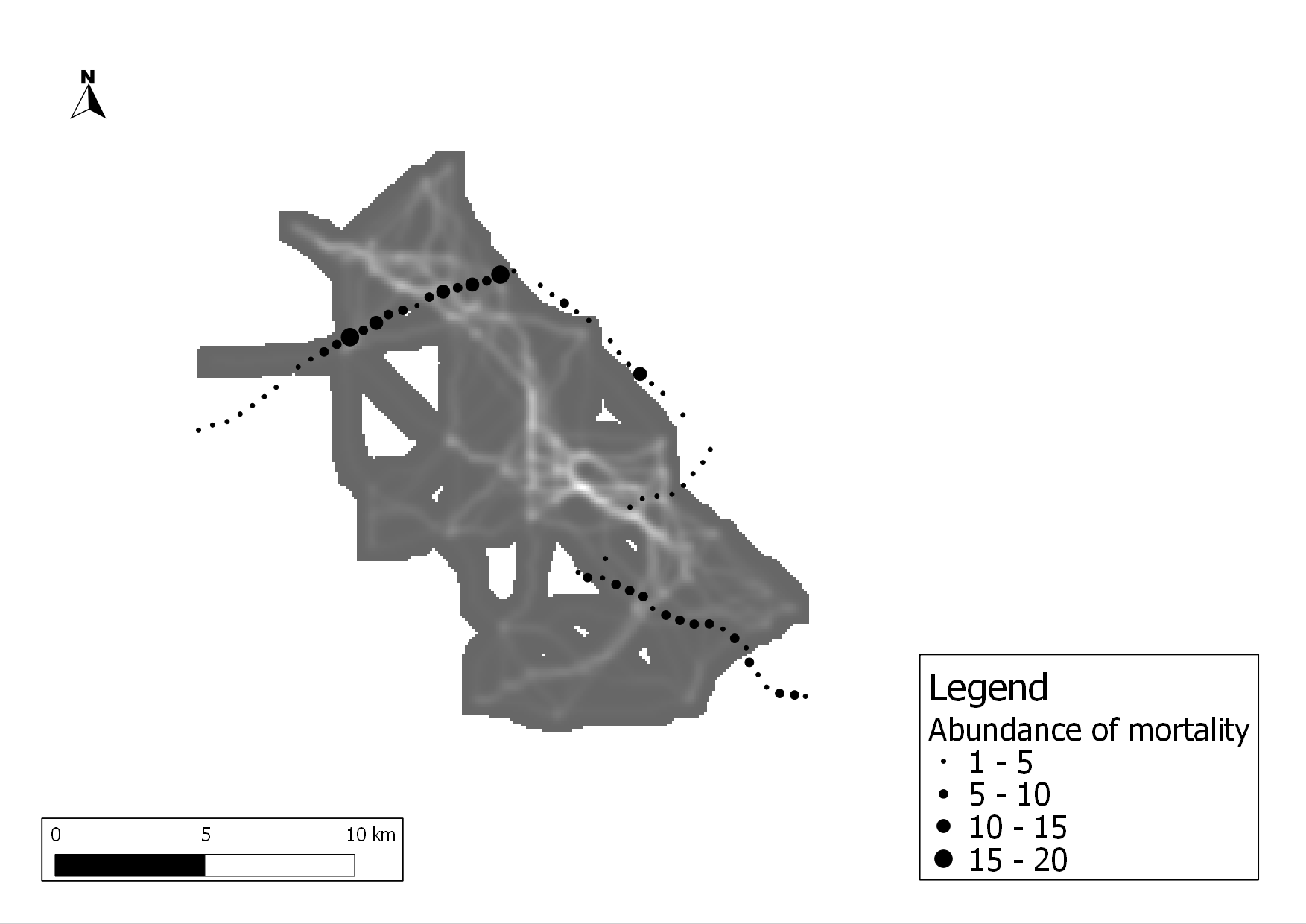

Supplement: Figure S7 — Connectivity model for a pattern of connectivity among high quality territories up to 5 km distance (HQ5), overlaid with owl mortality (lighter areas indicate higher movement probability). (TIF) [file pone.0079967.s007.tif]

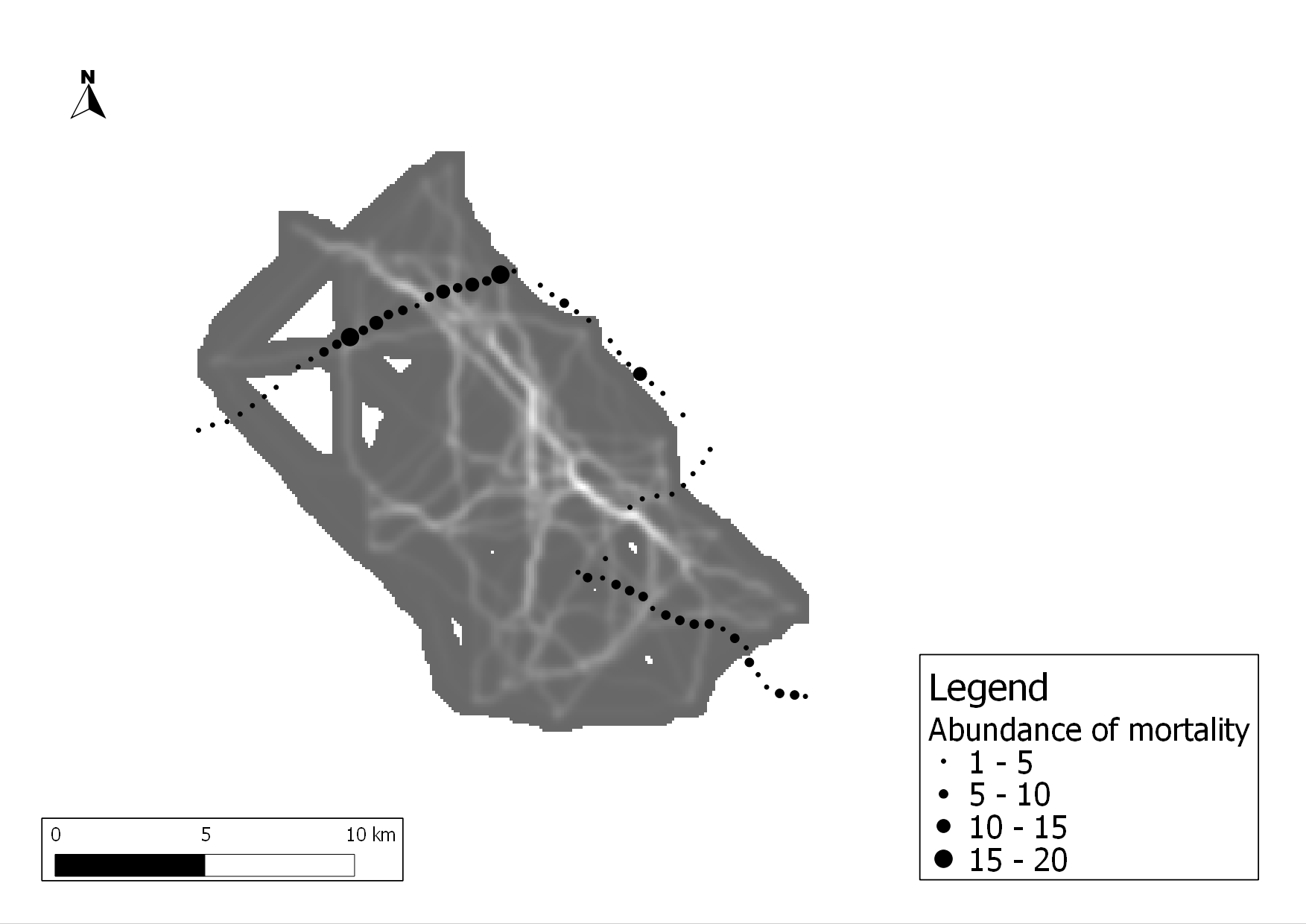

Supplement: Figure S8 — Connectivity model for a pattern of connectivity among high quality territories up to 10 km distance (HQ10), overlaid with owl mortality (lighter areas indicate higher movement probability). (TIF) [file pone.0079967.s008.tif]

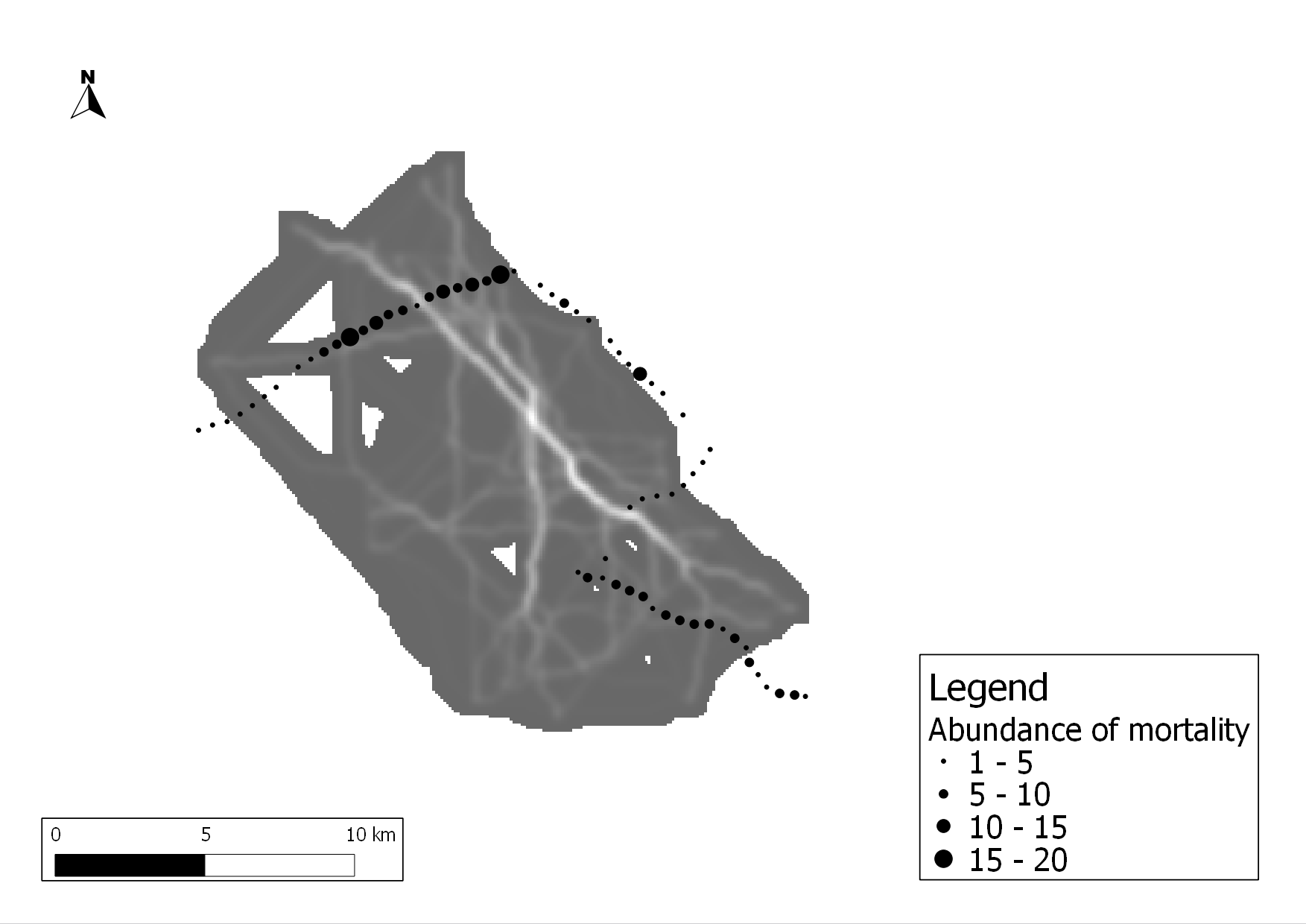

Supplement: Figure S9 — Connectivity model for a pattern of connectivity among high quality territories up to 100 km distance (HQ100), overlaid with owl mortality (lighter areas indicate higher movement probability). (TIF) [file pone.0079967.s009.tif]

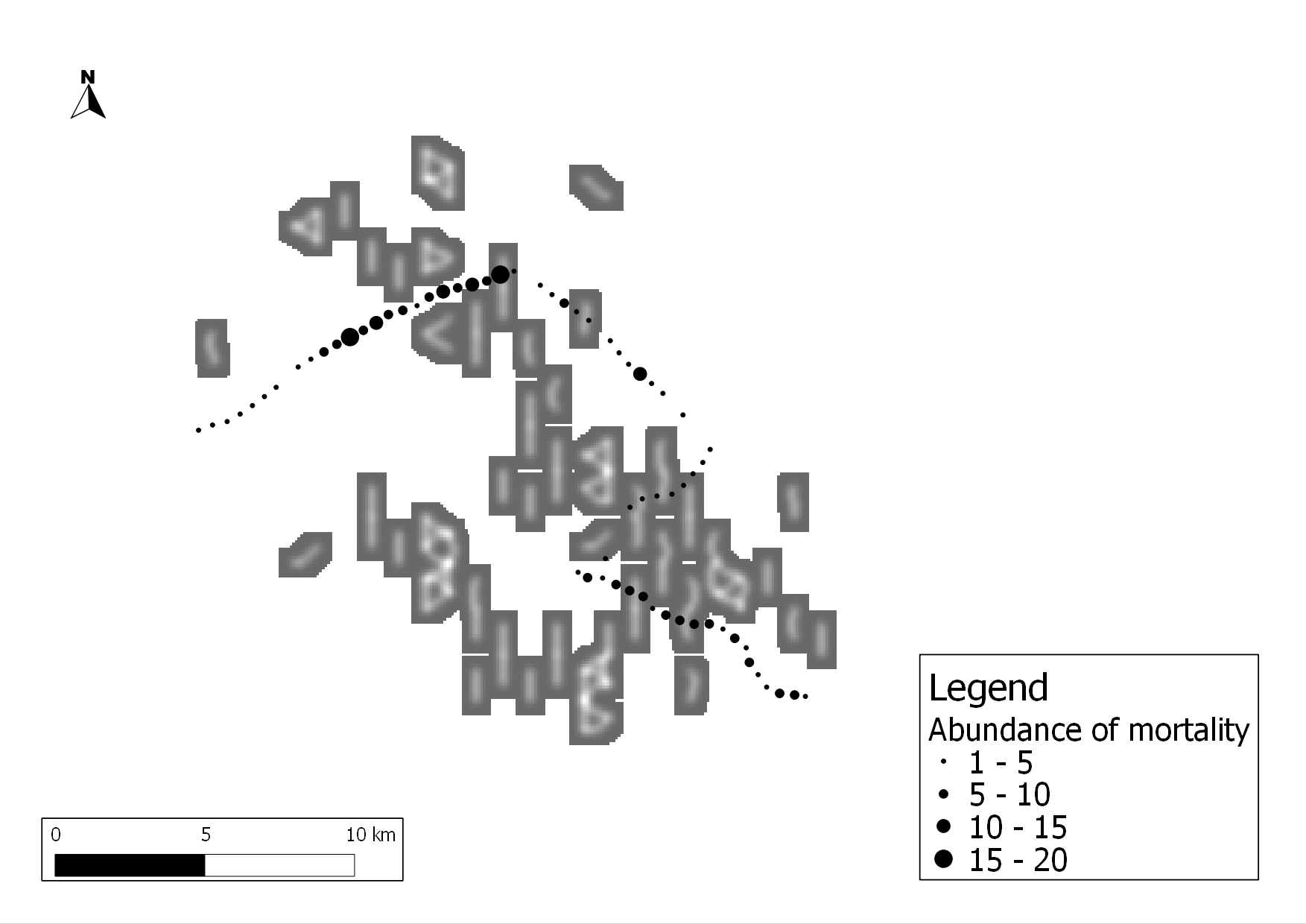

Supplement: Figure S10 — Connectivity model for a pattern of connectivity among favourable territories up to 1 km distance (F1), overlaid with owl mortality (lighter areas indicate higher movement probability). (TIF) [file pone.0079967.s010.tif]

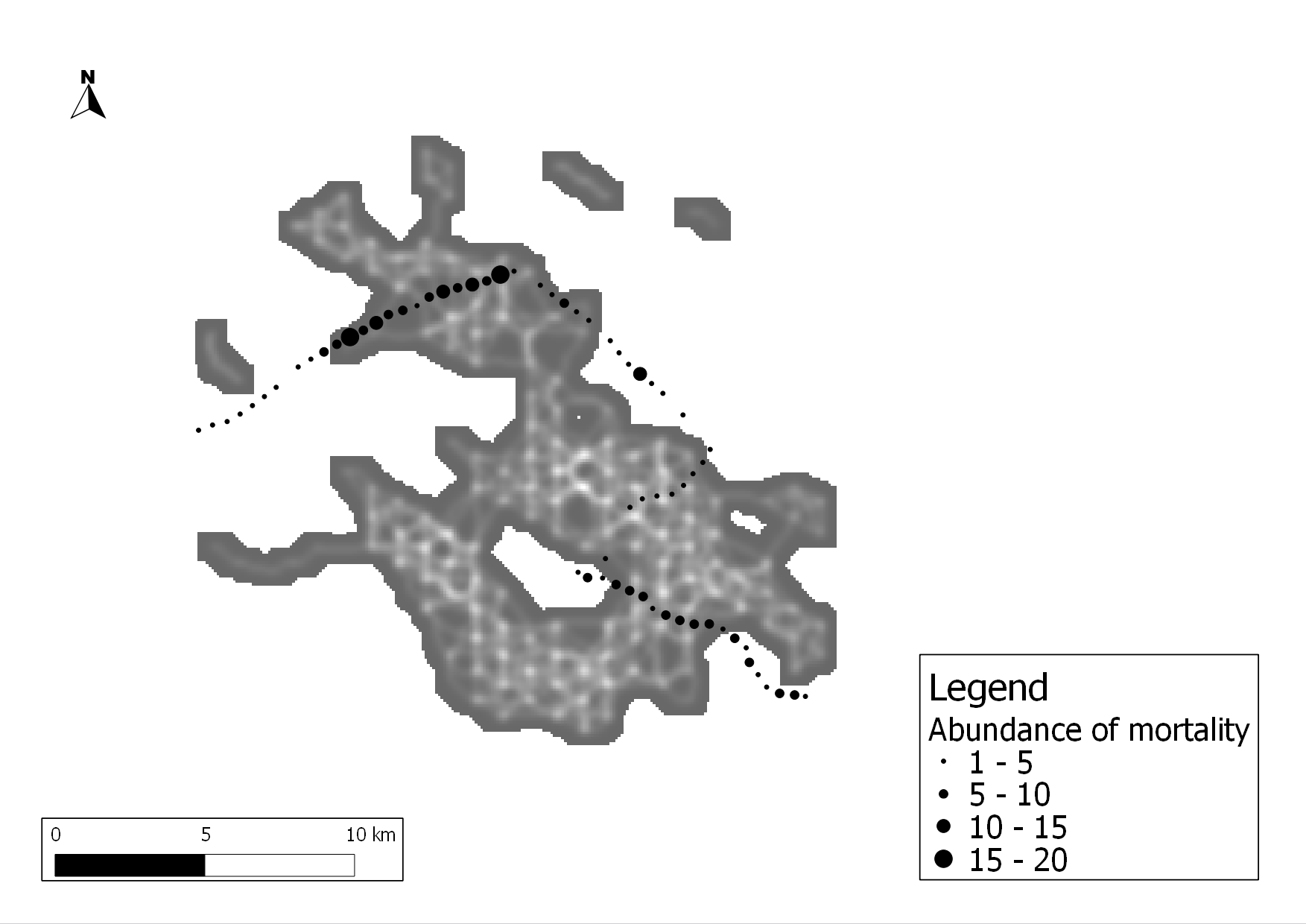

Supplement: Figure S11 — Connectivity model for a pattern of connectivity among favourable territories up to 2 km distance (F2), overlaid with owl mortality (lighter areas indicate higher movement probability). (TIF) [file pone.0079967.s011.tif]

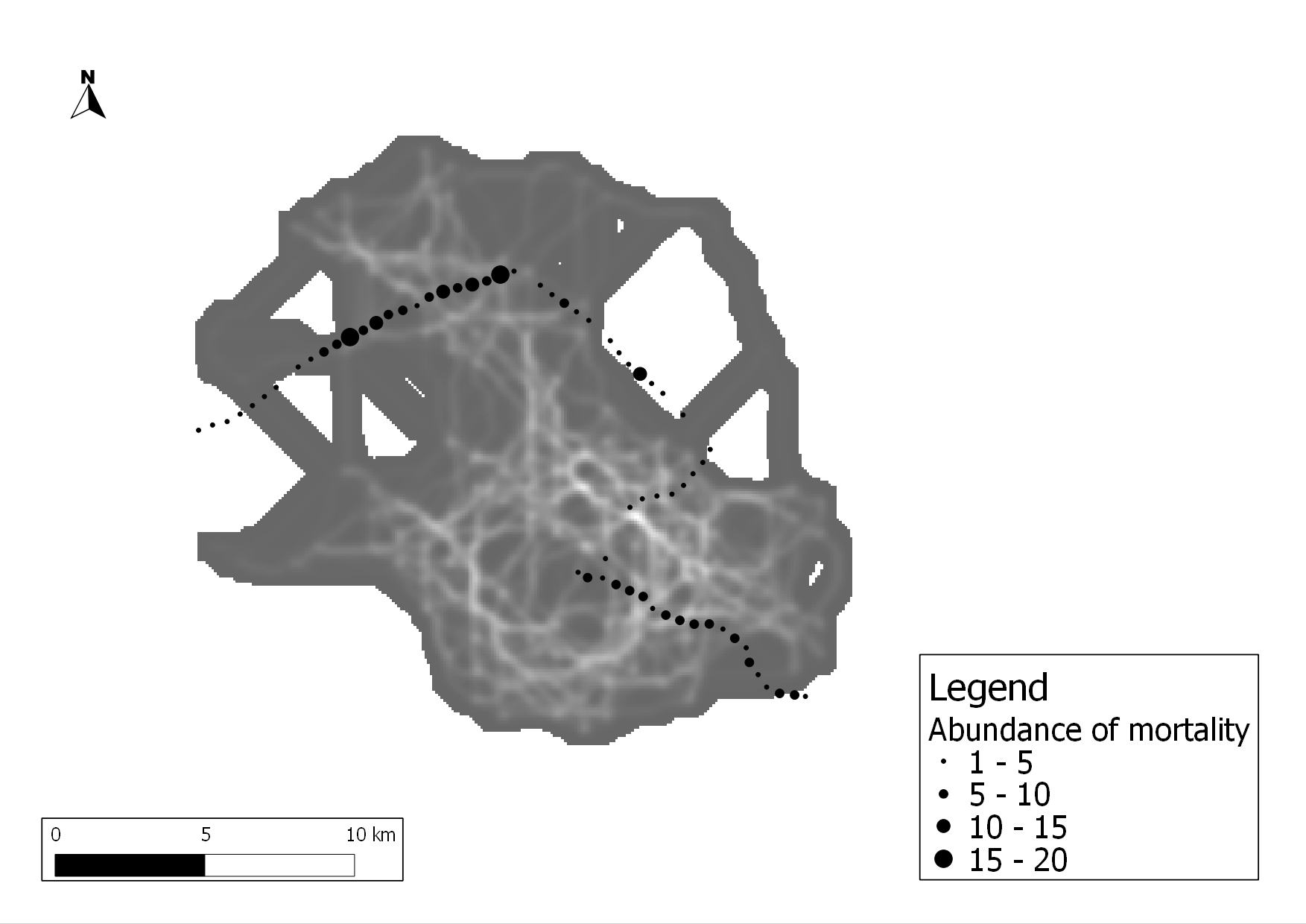

Supplement: Figure S12 — Connectivity model for a pattern of connectivity among favourable territories up to 5 km distance (F5), overlaid with owl mortality (lighter areas indicate higher movement probability). (TIF) [file pone.0079967.s012.tif]

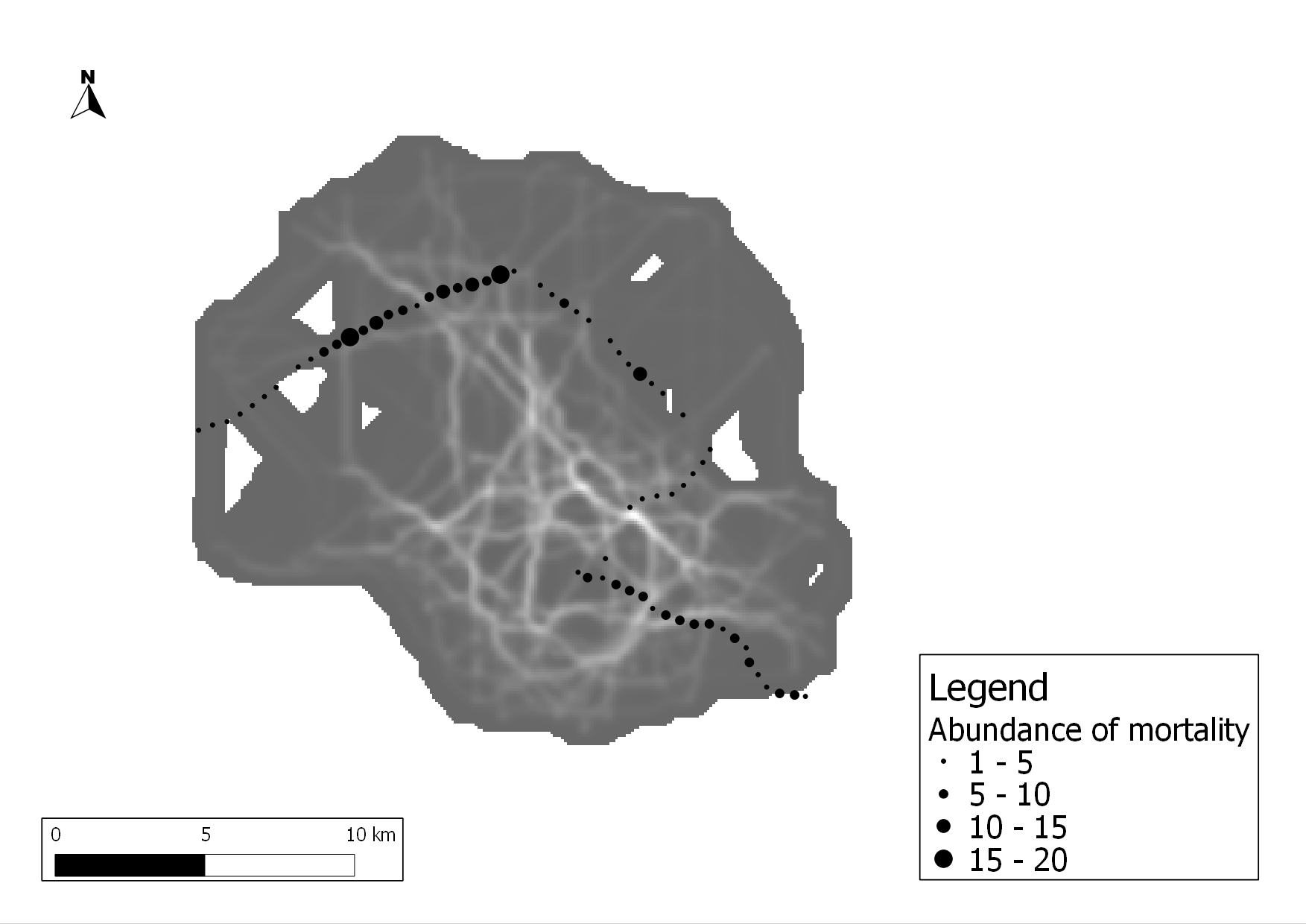

Supplement: Figure S13 — Connectivity model for a pattern of connectivity among favourable territories up to 10 km distance (F10), overlaid with owl mortality (lighter areas indicate higher movement probability). (TIF) [file pone.0079967.s013.tif]

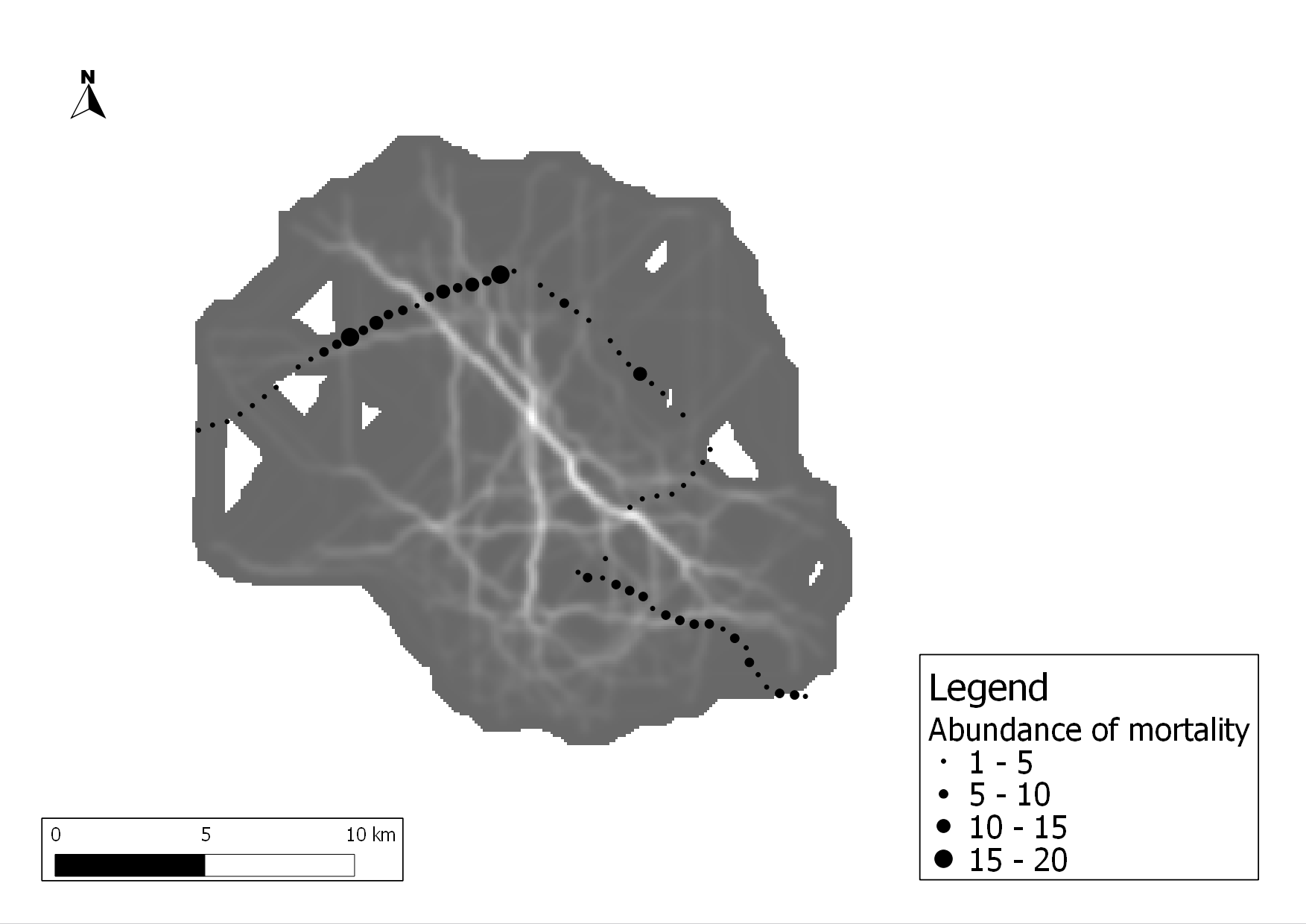

Supplement: Figure S14 — Connectivity model for a pattern of connectivity among favourable territories up to 100 km distance (F100), overlaid with owl mortality (lighter areas indicate higher movement probability). (TIF) [file pone.0079967.s014.tif]
